# The lithium effect in ketenyl anion chemistry†

**DOI:** 10.1039/d4cc03167a

**Published:** 2024-08-02

**Authors:** Prakash Duari, Sunita Mondal, Mike Jörges, Viktoria H. Gessner

**Affiliations:** a Faculty of Chemistry and Biochemistry, Ruhr-University Bochum 44801 Bochum Germany viktoria.gessner@rub.de

## Abstract

Ketenyl lithium compounds of type [RC(Li)CO] (with R = Ph_2_P(E), E = O, S, Se) were found to exhibit lower thermal stabilities than their potassium analogues due to the stronger coordination of the oxygen of the ketene moiety to the harder metal cation, resulting in a more pronounced ynolate character. Using additional ligands allows manipulation of the O–Li interaction, thereby influencing the stability and reactivity of the ketenyl anions.

## Abstract 2

Ketenyl lithium compounds were found to be less thermally stable than their potassium analogues due to the stronger binding of the oxygen of the ketene moiety to the lithium ion, resulting in a more pronounced ynolate character.

## Graphical abstract

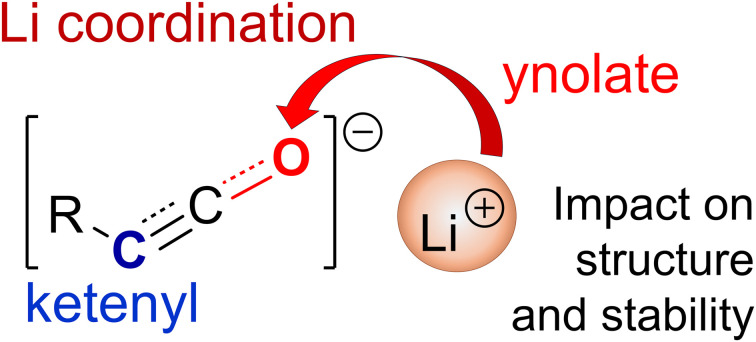

Ketenes are important intermediates in synthetic organic chemistry, offering access to a variety of carbonyl containing compounds.^1^ In general, ketenes are highly reactive species due to their polarized cumulated double bond.^2^ Therefore, they are often generated *in situ* and directly converted to the desired products. Over the years, several synthetic methods to ketenes have been developed, including the Wolff rearrangement of diazoketones,^3^ photolytic or pyrolytic reactions of suitable precursors,^4^ or the carbonylation of carbenes or metal carbenes.^5^ However, many of these methods have drawbacks, such as requiring harsh conditions or unstable starting materials, resulting in low yields and poor selectivity, or having limitations regarding substrate scope.

Ketenyl anions, [RC=C=O]^−^, offer an alternative entry into ketene chemistry. In the past, these anions were synthesized by carbonylation of diazomethanides (*e.g.*I, Fig. 1A) and used without isolation.^6,7^ Recently, we developed a novel synthetic route to ketenyl anions through a mild PPh_3_/CO exchange at the ylidic carbon center in α-metalated ylides.^8,9^ This method enabled the first isolation and structure elucidation of a ketenyl anion, revealing an intermediate bonding situation in the anion between the ketenyl and ynolate form (Fig. 1C). Using this synthetic pathway, we isolated the complete series of phosphinoyl-substituted ketenyl anions V,^10^ the tosyl-ketenyl anion (VI),^11^ and the cyano-substituted ketenyl anion (VII).^12^ Furthermore, the Liu group reported the isolation of ketenyl anion IIX using the N_2_/CO exchange in diazomethanides.^13^

**Fig. 1:**
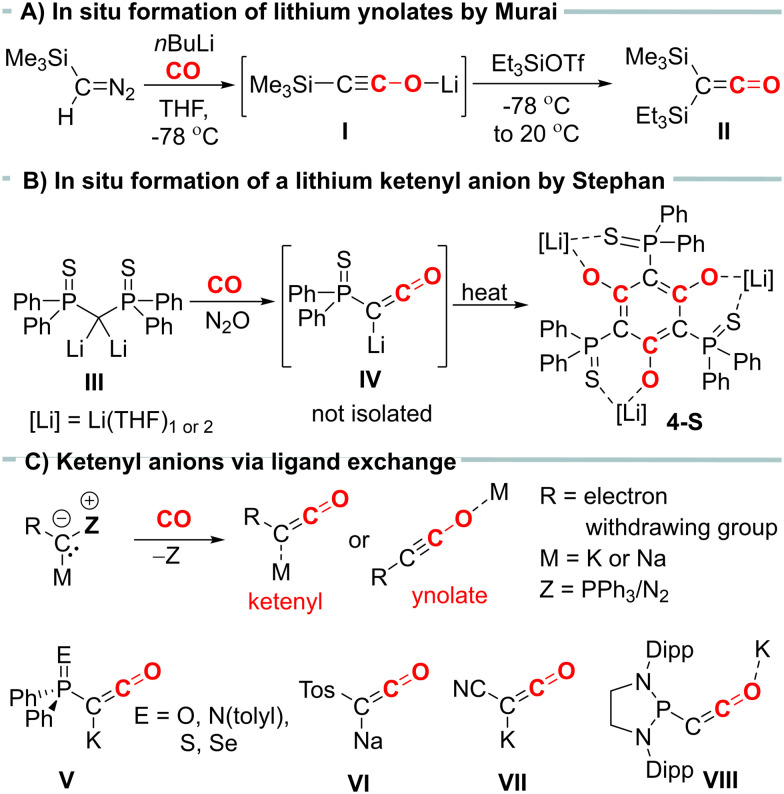
(A) *In situ* formation of a lithium ynolate by carbonylation of metalated a diazomethane. (B) Formation of ketenyl anion from the reaction of a dilithiomethandiide with CO and N_2_O or (C) *via* PPh_3_/CO exchange and examples of isolated ketenyl anions.

Remarkably, all isolated ketenyl anions reported to date have had either sodium or potassium as counter cation. No lithium salt has been isolated yet.^14^ In 2021, Stephan and coworkers reported the formation of Li[Ph_2_P(S)CCO] (IV) from the reaction of dilithiomethandiide III with CO and N_2_O (Fig. 1B),^15^ but in contrast to its potassium analogue V, it was unstable and cyclotrimerized to compound 4-S. Thus, we became interested in understanding the effect of lithium on the stability of ketenyl anions. We recently demonstrated that changes in the electronegativity of the substituents at carbon affect the relative preference of the ynolate *versus* the ketenyl form.^10^ Therefore, we hypothesized that a similar shift can be induced by the metal cation depending on the strength and site (C *versus* O) of the cation–anion interaction.

We began our investigations with the preparation of lithium yldiides 2 with different phosphinoyl moieties P=E (E = O, S, Se), the potassium analogues of which we have recently reported.^10^ Lithium diisopropylamide (LDA) was found to be a suitable base to selectively generate the α-metalated ylides 2 (Scheme 1).^15^ Due to their reactivity, yldiides 2 were not isolated but used as *in situ* prepared reagents. The formation of 2 was confirmed in each case by ^31^P{^1^H} NMR spectroscopy, which showed a significant high-field shift of the signal compared to the parent ylides 1 (see ESI†).^16^ For 2b and 2c, few crystals suitable for X-ray diffraction (XRD) analysis were obtained by the slow diffusion of hexane into toluene/THF solutions, further confirming the successful metalation.

**Scheme 1:**
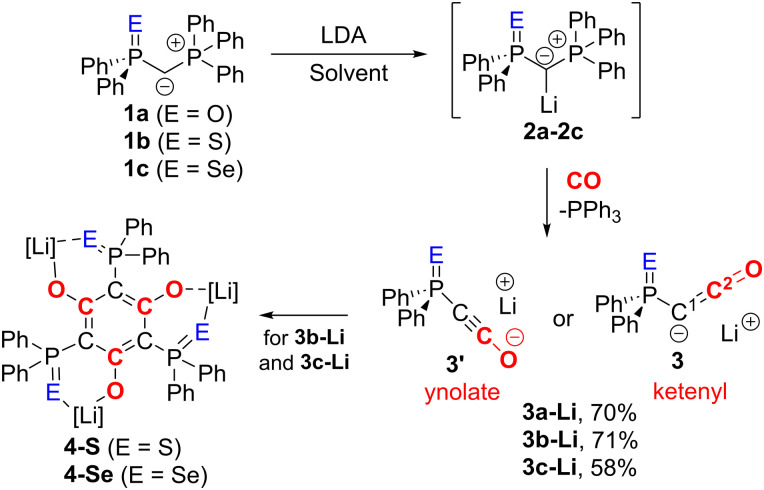
Synthesis of yldiide 2, ketenyl anion 3 and their decomposition products 4. Solvent = toluene (for E = O), toluene/THF mixture (E = S, Se).

In the crystal, the metalated ylides 2b and 2c form centrosymmetric dimers, with the lithium cations being coordinated by the S and Se atom, respectively, the ylidic carbon atom, and an additional THF molecule (see Fig. 2 for 2b, the ESI† for 2c). In 2b, the P–C distances (1.671(2) and 1.651(2) Å) are significantly shorter than in the ylide precursor (1.729(1) and 1.696(1) Å) due to increased electrostatic interactions within the P–C–P linkage upon metalation. A similar trend has been observed for the corresponding potassium compound with 18-crown-6.^17^

**Fig. 2:**
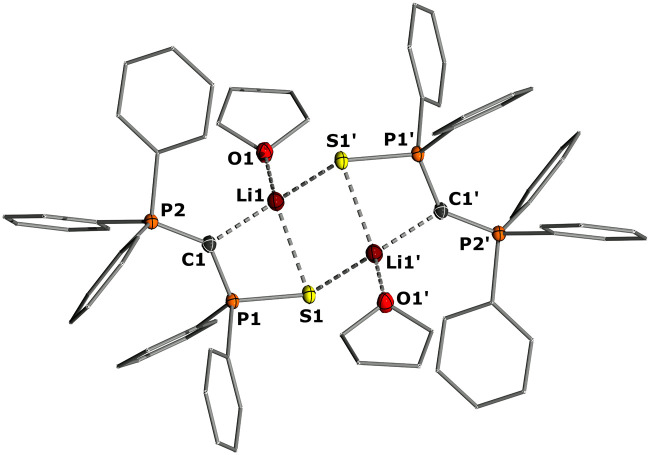
Crystal structure of 2b (ellipsoids at the 50% probability level, hydrogen atoms omitted for clarity). Selected bond lengths [Å] and angles [°]: P1–C1 1.671(2), P2–C1 1.651(2), P1–S1 2.030(1), C1–Li1 2.128(3), S1–Li1 2.558(3), P1–C1–P2 135.9(1).

Once the selective formation of the metalated ylides 2 was confirmed, we examined their reactivity towards CO and selected 2c as first test system. Its reaction with CO in toluene/THF solution led to the selective formation of PPh_3_, indicated by a signal at −5.26 ppm in the ^31^P{^1^H} NMR spectrum, and another species characterized by a signal at 5.57 ppm, which we assigned to the targeted lithium ketenyl 3c-Li. 3c-Li could be isolated as a colourless solid in 58% yield. The lithium ketenyl is further characterized by a doublet at 5.9 ppm with ^1^*J*_PC_ coupling constant of 184.9 Hz for the C1 carbon atom and another doublet at 139.8 ppm with ^2^*J*_PC_ coupling constant of 45.0 Hz for the C2 carbon atom in ^13^C{^1^H} NMR spectrum (Table 1). The larger *J*_PC_ coupling constants of the C1 and C2 carbon atom compared to those in the potassium analogue 3c-K (164.0 and 39.0 Hz) indicate a larger P–C–C angle and the preference for the ynolate form in 3c-Li relative to 3c-K.^18^

**Table 1:** NMR spectroscopic data of the lithium salts of the ketenyl anions compared with their potassium salts. NMR Shifts are given in ppm and coupling constants in Hz

	3a-Li	3a-K	3b-Li	3b-K	3c-Li	3c-K
*δ*(P)	12.3	13.2	21.6	22.5	5.6	6.2
*δ*(C1)	2.3	3.1	6.7	2.4	5.9	2.5
^1^ *J*_PC_	218.3	210.0	193.8	175.0	184.9	164.0
*δ*(C2)	141.9	140.8	138.3	142.7	139.8	143.9
^2^ *J*_PC_	48.8	47.5	46.0	40.7	45.0	39.0

Interestingly, lithium ketenyl 3c-Li was found to be unstable in solution. At room temperature, a THF solution converted to a new compound within a few days. This compound was identified as the hexa-substituted benzene derivative 4-Se, formed by trimerization of the ketenyl anion (see ESI†). This trimerization is consistent with the observation made by the Stephan group (Fig. 1B) but contrasts the stability of the potassium analogue of IV, which showed no analogous trimerization reaction at room temperature.^15^

Given the lower stability of the lithium salt 3c-Li relative to its potassium salt 3c-K, we next evaluated whether this was a general trend. To this end, the lithium ketenyls 3a-Li and 3b-Li were isolated as colourless solids in high yields of over 70% after stirring of the metalated ylide in toluene/THF solution under 1 atm. of CO overnight (Scheme 1). Like the selenide, 3a-Li and 3b-Li show two characteristic doublets in the ^13^C{^1^H} NMR spectrum for the C1 and C2 carbon atom, with ^1^*J*_PC_ and ^2^*J*_PC_ coupling constants larger than those observed for the corresponding potassium compounds (Table 1). Thus, the oxide and the sulfide confirm the trend observed for the selenide, further suggesting that the lithium compounds exhibit a more pronounced ynolate character. Nonetheless, 3b-Li was found to be surprisingly stable in THF solution at room temperature, but it began to trimerize to 4-S when heated to 70 °C. Full conversion was observed after 7 days (Fig. 3). The phosphine oxide 3a-Li demonstrated even greater stability. After heating a THF solution of 3a-Li at 70 °C for 2 weeks, no noticeable changes were observed in the ^31^P{^1^H} NMR spectrum. These observations suggest that increased ynolate character generally decreases the stability of the lithium compounds, but this trend varies with different substituents. According to the *J*_PC_ coupling constants, the ynolate character increases from the phosphine selenide to the oxide, yet the stability increases in the same order. We assumed that this contradictory trend is due to lithium binding to both the ketenyl and the phosphinoyl moiety. The strength of the E–Li interaction increases with the hardness of the donor (O > S > Se), leading to a greater electron density shift towards the Group 16 element. Thus, the *J*_PC_ coupling constant increases due to a stronger C–P(E) bond, rather than an increased ynolate character.

**Fig. 3:**
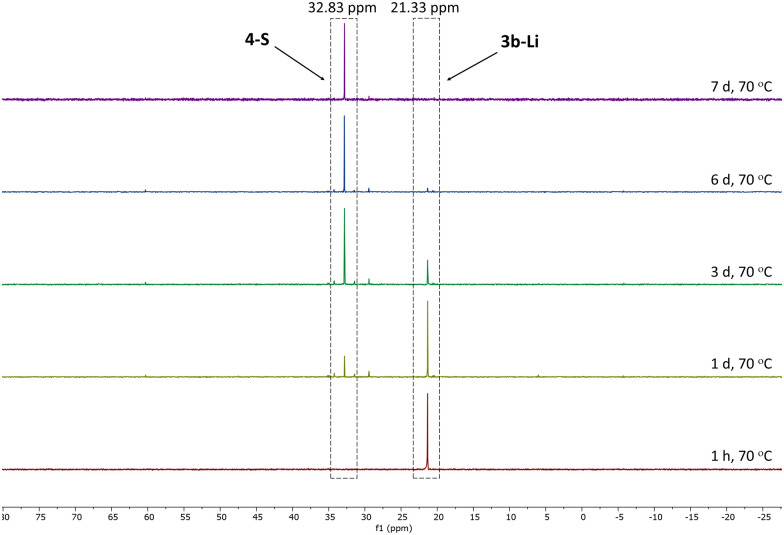
Monitoring of the conversion of 3b-Li into 4-S by ^31^P{^1^H} NMR spectroscopy in THF-d_8_ at 70 °C.

To gain structural insights into the ketenyl lithium compounds, we attempted to obtain single crystals for XRD analysis. Suitable crystals of 3a-Li were grown by slow diffusion of hexane into a saturated toluene/THF solution (Fig. 4, top). In the solid state, 3a-Li forms a polymeric structure with a Li_2_O_2_ four-membered ring as central structural motif.^19^ The lithium ions are each coordinated by the phosphinoyl moiety of two molecules of 3a, the ketenyl oxygen of two neighboring molecules and an additional THF molecule. No contact to the ketenyl C1 carbon atom is observed. The C1–C2 bond in the ketene moiety is slightly shorter (∼1.230 Å) and C–O bond (∼1.214 Å) is slightly longer than in the potassium salt 3a-K with [2,2,2]-cryptand (1.240 Å for C1–C2 and 1.212 Å for C2–O), indicating a stronger contribution of the ynolate form 3′ in the lithium salt as suggested by the *J*_PC_ coupling constants.^10^ Accordingly, the P–C–C angle (∼160.5°) in 3a-Li is also larger than in the potassium salt (148.9°), almost approaching linearity.

**Fig. 4:**
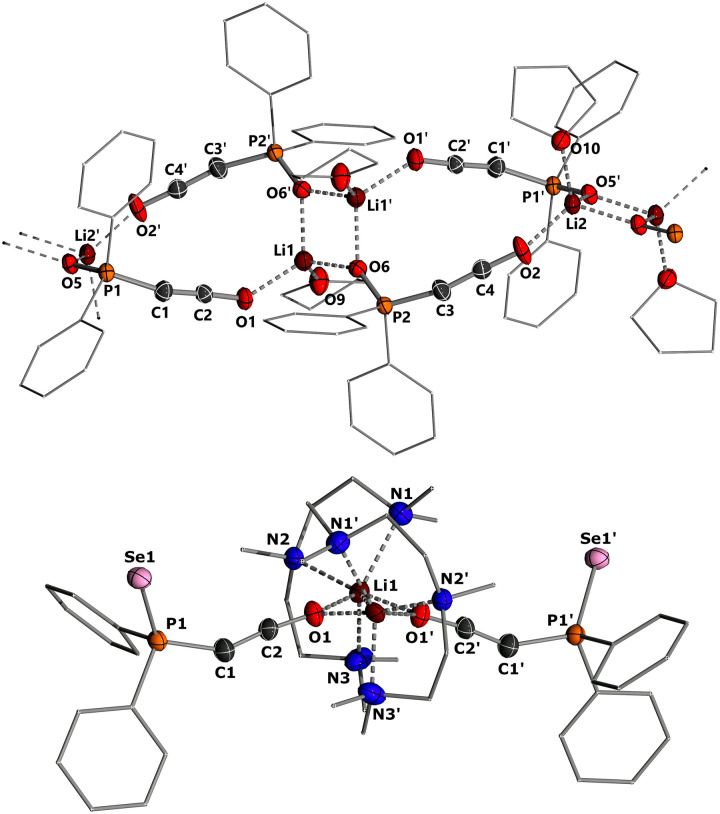
Crystal structure of (top) 3a-Li and (bottom) 3c-Li(PMDETA). Ellipsoids are drawn at the 50% probability level. Important bond lengths [Å] and angles [°]: 3a-Li: P1–C1 1.677(4), C1–C2 1.230(6), C2–O1 1.216(5), P1–O1 1.510(3), P1–C1–C2 161.9(4), C1–C2–O1 176.7(5), P2–C3 1.673(4), C3–C4 1.231(6), C4–O2 1.206(5), P2–O6 1.509(3), P2–C3–C4 162.4(4), C1–C2–O1 176.7(5). 3c-Li(PMDETA): P1–Se1 2.132(1), P1–C1 1.698(6), C1–C2 1.243(8), C2–O1 1.222(7), O1–Li1 2.069(10), P1–C1–C2 147.8(5), C1–C2–O1 173.9(6).

Since we could not obtain suitable crystals for the THF complexes of 3b-Li and 3c-Li, we isolated the lithium salts 3 with PMDETA (*N*,*N*,*N*′,*N*′′*N*′′-pentamethyldiethylenetriamine)^20^ as additional ligand to gain further information about the coordination chemistry of the lithium ketenyls.^21^ We hypothesized that the coordination of PMDETA would weaken the O–Li interaction and thereby increase stability. Stirring of the α-metalated ylides 2 with 1 eq. of PMDETA in toluene/THF solvent under an atmosphere of CO led to the precipitation of the corresponding complexes 3-Li(PMDETA), which were isolated as colourless solids in moderate to good yields (see the ESI†). In contrast to the THF complexes, all PMDETA complexes turned out to be stable and did not trimerize to the corresponding benzene derivatives 4, demonstrating the advantageous influence of the ligand on stability. Single crystals of 3c-Li(PMDETA) were obtained by slow diffusion of hexane into a toluene/THF solution at −30 °C. In the solid-state, 3c-Li(PMDETA) forms a centrosymmetric dimer with the lithium cations coordinated by PMDETA and the oxygen of the ketenyl moiety (Fig. 4, bottom). The C–C and C–O bond lengths in the ketene moiety (1.243(8) and 1.222(7) Å) slightly change towards the values of the free anion (1.244(5) and 1.207(4) Å),^10^ indicating that the additional ligand weakens the O–Li interaction, thereby increasing the ketenyl character in C–C–O moiety and enhancing its stability.

Given that the structural changes are minimal, we aimed to further confirm that the strength of the lithium–oxygen interaction affects the bonding situation by examining the IR vibration of the C=C=O group. Generally, the IR stretch of ketenyl anions falls between those of ketenes and alkynyl ethers,^22^ with the vibration of ketenes (*e.g.*, 2080 cm^−1^ for (SiR_3_)(PhCH_2_CH_2_)C=C=O) appearing distinctly red-shifted compared to those of alkynyl ethers (*e.g.*, 2280 cm^−1^ for PhCH_2_CH_2_–C≡C–OSiMe_3_).^23,24^ Thus, the C=C=O stretch is highly diagnostic for determining the ketenyl and ynolate character. IR measurements of anions 3-Li, with and without PMDETA, were performed in the solid state as well as in THF solution to exclude solid-state effects (Table 2). In solid state, the lithium ketenyls without PMDETA exhibit stretching frequencies higher than 2125 cm^−1^. In contrast, their potassium analogues show frequencies lower than 2100 cm^−1^, thus corroborating with a more pronounced ynolate character of the lithium ketenyls. In addition, the value for the phosphine oxide 3a-Li is lower than those of the sulfide (3b-Li) and selenide (3c-Li), corroborating with its higher stability. The stretching frequencies of 3-Li with PMDETA are lower than those without it, corroborating with a shift towards increased ketenyl character and the enhanced stability of the ketenyl anions in presence of the additional ligand. In THF solution, the frequencies were found to be independent of the presence or absence of the PMDETA ligand, likely due to decreased cation–anion interactions in the coordinating THF solvent.

**Table 2:** IR stretching frequencies of the ketenyl moiety in the lithium ketenyl complexes 3 with and without PMDETA (in cm^−1^)

	Solid state		THF solution	
	Ligand-free	+PMDETA	Ligand-free	+PMDETA
3a-Li	2129.83	2109.72	2093.91	2093.92
3b-Li	2142.03	2103.26	2126.31	2125.52
3c-Li	2138.44	2098.23	2124.80	2124.08

In conclusion, we have isolated lithium salts of phosphinoyl-substituted ketenyl anions through carbonylation of lithium yldiides through elimination of PPh_3_. These lithium ketenyl anions are less thermally stable towards trimerization than their potassium analogues. NMR and IR spectroscopy as well as crystallographic studies showed that this lower stability is accompanied by lithium's preferential coordination by the oxygen atom of the ketene moiety. This coordination favours the ynolate character, indicated by larger coupling constants, blue-shifted C–C–O vibrations, and shorter C–C bonds. The bonding situation can be further influenced by using an additional ligand like PMDETA, weakening the Li–O interaction. These findings highlight the flexibility of the bonding situation in ketenyl anions, which can be regulated through ion-pairing. These effects decisively impact the stability of the anions and can be utilized for reactivity control essential for future applications.

This work was supported by RESOLV, funded by the Deutsche Forschungsgemeinschaft (DFG, German Research Foundation) under Germany's Excellence Strategy – EXC-2033 – project number 390677874 and by the European Research Council (Starting Grant: YlideLigands 677749). P. D. acknowledges the German Academic Exchange Service for a PhD scholarship.

## Data availability

The data supporting this article have been included as part of the ESI.†

## Conflicts of interest

There are no conflicts of interest.

## Supplementary Material

CC-060-D4CC03167A-s001

CC-060-D4CC03167A-s002
