# Supplementary material for: The lithium effect in ketenyl anion chemistry
Source: Chem Commun (Camb). 2024 Aug 2;60(70):9372–5. doi: 10.1039/d4cc03167a (PMC11322804; doi:10.1039/d4cc03167a)
Supplement: CC-060-D4CC03167A-s001 [file CC-060-D4CC03167A-s001.pdf]

Supplementary Information (SI) for ChemComm.
This journal is © The Royal Society of Chemistry 2024

Supporting Information

The Lithium Effect in Ketenyl Anion Chemistry

Prakash Duari,[a] Sunita Mondal,[a] Mike Jörges,[a] and Viktoria H. Gessner*[a]

[a]Faculty of Chemistry of Biochemistry, Ruhr-University Bochum, 44801 Bochum, Germany, Email: viktoria.gessner@rub.de

Table of Contents

1. Experimental Procedures ... 2
- 1.1. General Information ... 2
- 1.2. Synthesis of compounds 2 ... 3
- 1.3. Synthesis of compounds 3-Li ... 4

2. NMR and IR spectra ... 8
- 2.1. NMR spectra of the metallated ylides 2 ... 8
- 2.2. NMR and IR spectra of compound 3a-Li ... 9
- 2.3. NMR and IR spectra of compound 3b-Li ... 15
- 2.4. NMR and IR Spectra of compound 3c-Li ... 21

3. Crystal structure determination ... 28
- 3.1. General information ... 28
- 3.2. Molecular structure of 2b ... 30
- 3.3. Molecular structure of 2c ... 31
- 3.4. Molecular structure of 3a-Li ... 31
- 3.5. Molecular structure of 3c-Li(PMDETA) ... 32
- 3.6. Molecular structure of 4c ... 32
- 3.7. Molecular structure of potassium ketenyl anions ... 33

4. Author contributions ... 34

5. References ... 35

1. Experimental Procedures

1.1. General Information

Chemical and Conditions

If not stated otherwise, all experiments were carried out using standard Schlenk techniques under an argon atmosphere, which was dry and free of oxygen. Argon (99.999%) was a product of *Air Liquide* and was used without any further drying. An MBraun SPS 800 was used to dry solvents before their usage (THF, toluene, DCM, ACN, *n*-pentane, *n*-hexane). All solvents were stored over molecular sieves under an argon atmosphere. Reagents were purchased from Sigma-Aldrich, ABCR, Acros Organics or TCI Chemicals and used without further purification if not stated otherwise. **1a**, **1b**, **1c** were synthesized following literature procedures.[1,2]

Analytical methods

NMR Spectroscopy. ^{1}H, $^{13}C\{^{1}H\}$, $^{77}Se\{^{1}H\}$, $^{31}P\{^{1}H\}$ NMR spectra were recorded on Avance-III-400 spectrometers at 22 °C if not stated otherwise. All values of the chemical shift are in ppm regarding the δ-scale. All spin-spin coupling constants (*J*) are printed in Hertz (Hz). To display multiplicities and signal forms correctly the following abbreviations were used: s = singlet, d = doublet, t = triplet m = multiplet, dd = doublet of doublet, br = broad signal. Signal assignment was supported by, HSQC (^{1}H / ^{13}C), HMBC (^{1}H / ^{13}C, ^{1}H / ^{31}P) correlation experiments.

IR spectra were recorded on a Shimadzu IRSpirit with QATR-S module in an argon filled glovebox and on a Thermo Nicolet iS5 FT-IR in transmission mode with a Specac “Omni-cell” with KBr plates and a 0.1 mm spacer at 22 °C. Measurement and processing details for individual spectra can be extracted from the corresponding tables in the supporting information.

Melting points were measured with the SMP30 melting point apparatus from Stuart.

Elemental analyses were performed on an Elementar vario MICRO cube elemental analyzer.

For details about the single-crystal Xray diffraction analyses, see chapter 3.

Safety comments

Caution! Strong bases such as organolithium bases, especially as neat compounds, are severely air-/moisture-sensitive and pyrophoric organometallic compounds. These compounds need to be handled under an inert gas atmosphere to exclude reactions with oxygen and water. Guidelines for their handling can be found in literature: T. L. Rathman, J. A. Schwindeman, *Org. Process Res. Dev.* **2014**, *18*, 1192.

Caution! Carbon monoxide (CO) is a highly toxic gas. Reactions should be performed in well-ventilated fumehoods, ideally with a CO sensor.

.

1.2. Synthesis of compounds 2

Synthesis of compounds 2a

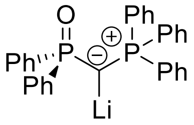

20 mg (0.0406 mmol) of compound **1a** and 4.93 mg (0.0447 mmol) LDA were dissolved in 1 mL THF-d_8 and stirred for 10 min. After filtration, a yellow solution of **2a** was obtained. The compound was not isolated but used as a solution for further reaction. The successful formation of **2a** was confirmed by $^1P\{^1H\}$-NMR spectroscopy.

$^{31}P\{^1H\}$-NMR (162 MHz, THF-d_8): δ = 31.83 (d, $^2J_{PP}$ = 75.2 Hz, *P*Ph_2O), -4.91 (d, $^2J_{PP}$ = 75.2 Hz, *P*Ph_3) ppm.

Synthesis of compounds 2b

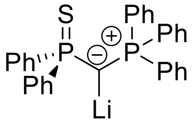

20 mg (0.0406 mmol) of compound **1b** and 4.93 mg (0.0447 mmol) LDA were dissolved in 1 mL THF-d_8 and stirred for 10 min. After filtration, a yellow solution of **2b** was acquired. The compound was not isolated but used as a solution for further reaction. The successful formation of **2b** was confirmed by $^1P\{^1H\}$-NMR spectroscopy. In addition, few crystals suitable for X-ray diffraction (XRD) analysis could be grown by slow diffusion of hexane into toluene/THF solution.

$^{31}P\{^1H\}$-NMR (162 MHz, THF-d_8): δ = 26.82 (d, $^2J_{PP}$ = 32.2 Hz, *P*Ph_2S), -11.88 (d, $^2J_{PP}$ = 32.2 Hz, *P*Ph_3) ppm.

Synthesis of compounds 2c

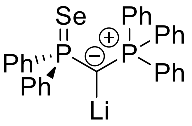

20 mg (0.0371 mmol) of compound **1c** and 4.51 mg (0.0408 mmol) LDA were dissolved in 1 mL THF-d_8 and stirred for 10 min. After filtration, a yellow solution of **2c**was acquired. The compound was not isolated but used as a solution for further reaction. The successful formation of **2a** was confirmed by $^1P\{^1H\}$-NMR spectroscopy. In addition, few crystals suitable for X-ray diffraction (XRD) analysis could be grown by slow diffusion of hexane into toluene/THF solution.

$^{31}P\{^1H\}$-NMR (162 MHz, THF-d_8): δ = 9.12 (d, $^2J_{PP}$ = 18.6 Hz, *P*Ph_2Se), -10.01 (d, $^2J_{PP}$ = 18.6 Hz, *P*Ph_3) ppm.

1.3. Synthesis of compounds 3-Li

Synthesis of compounds 3a-Li

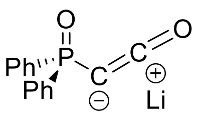

1.5 g (3.15 mmol) of **1a** and 382.0 mg (03.46 mmol) of LDA were dissolved in 30 mL tol/thf (9:1) mixture. The solution was stirred for 20 min. After filtration the atmosphere in the flask was changed from argon to CO and the reaction was stirred for 24 hours. After concentrating the solution to 5ml, 30 mL pentane was added resulting in the precipitation of a solid. The solid was filtered and washed with toluene (3 times) and pentane (3 times). After drying in vacuo, lithium ketenyl **3a-Li** was obtained as off-white solid. (702 mg, 3.15 mmol, 70 %).

^{1}H-NMR (400 MHz, THF-d_8): δ = 7.93 – 7.80 (m, 4H, $PPh_2OHPh,ortho$), 7.29 – 7.18 (m, 6H, $PPh_2OHPh,meta,para$), 3.65 – 3.59 (m, 2H, THF-OCH_2), 1.80 – 1.74 (m, 2H, THF-OCH$_2$CH_2). **^{13}C{^{1}H}-NMR** (101 MHz, THF-d_8): δ = 142.8 (d, $^{1}J_{PC}$ = 120.3 Hz, $PPh_2OC_{Ph,ipso}$), 141.91 (d, $^{2}J_{PC}$ = 48.8 Hz, PC*C*O), 131.93 (d, $^{2}J_{PC}$ = 10.4 Hz, $PPh_2OC_{Ph,ortho}$), 130.04 (d, $^{4}J_{PC}$ = 2.8 Hz, $PPh_2OC_{Ph,para}$), 128.24 (d, $^{3}J_{PC}$ = 12.4 Hz, $PPh_2OC_{Ph,meta}$), 68.39 (s, THF-OCH$_2$CH$_2$), 26.54 (s, THF-OCH$_2$CH$_2$), 2.28 (d, $^{1}J_{PC}$= 218.3 Hz, P*C*CO) ppm. **^{7}Li NMR** (156 MHz, THF-d_8) δ = -1.92 ppm. **^{31}P{^{1}H}-NMR** (162 MHz, THF-d_8): δ = 12.26 (s, *P*Ph$_2$O) ppm. **FT-IR** (ATR, cm^{-1}): 3058.3 (bw), 2977.9 (bw), 2873.8 (bw), 2129.8 (s, CCO stretching), 1483.5 (w), 1435.4 (s), 1127.4 (m), 1118.1 (s), 1082.9 (s), 1067.8 (m), 1053.4 (s), 1027.6 (w), 912.0 (bw), 715.2(s), 692.9 (s), 650.6 (m), 616.8 (w), 550.8 (m), 528.5(s). **Anal. Calcd.** for $C_{18}H_{18}Li_1O_3P_1$: C, 67.51; H, 5.67. Found: C, 67.24; H, 5.49.

PMDETA complex of 3a-Li

1.5 g (3.15 mmol) of **1a** and 382.0 mg (03.46 mmol) of LDA were dissolved in 30 mL tol/thf (9:1) mixture. The solution was stirred for 20 min. 1.1 eq. of *N,N,N',N"N"*-pentamethyl-diethylenetriamine (PMDETA) (606 mg, 3.46 mmol) was added to the reaction mixture. After filtration the atmosphere in the flask was changed from argon to CO and the reaction was stirred for 24 hours. After concentrating the solution to 5ml, 30 mL pentane was added resulting in the precipitation of a solid. The solid was filtered and washed with toluene (3 times) and pentane (3 times). After drying in vacuo, **3a-Li**(PMDETA) was obtained as off-white solid (938 mg, 2.23 mmol, 70.7 %).

^{1}H-NMR (400 MHz, THF-d_8): δ = 7.95 – 7.76 (m, 4H, $PPh_2OHPh,ortho$), 7.32 – 7.13 (m, 6H, $PPh_2OHPh,meta,para$), 2.44 (t, $^{3}J_{HH}$ = 6.7 Hz, 4H, CH_2-PMDETA), 2.34 (t, $^{3}J_{HH}$ = 6.7 Hz, 4H, CH_2-PMDETA), 2.25 (s, 3H, CH_3-PMDETA), 2.19 (s, 12H, CH_3-PMDETA). **^{13}C{^{1}H}-NMR** (101 MHz, THF-d_8): δ = 142.9 (d, $^{1}J_{PC}$ = Hz, $PPh_2OC_{Ph,ipso}$), 142.0 (d, $^{2}J_{PC}$ = 48.5 Hz, PC*C*O), 131.8 (d, $^{2}J_{PC}$ = 10.4 Hz, $PPh_2OC_{Ph,ortho}$), 129.9 (d, $^{4}J_{PC}$ = 2.8 Hz, $PPh_2OC_{Ph,para}$), 128.1 (d, $^{3}J_{PC}$ = 12.3 Hz, $PPh_2OC_{Ph,meta}$), 58.7 (s, *C*H$_2$-PMDETA), 56.8 (s, *C*H$_2$-PMDETA), 46.3 (s, *C*H$_3$-PMDETA), 43.7 (s, *C*H$_3$-PMDETA), 2.2 (d, $^{1}J_{PC}$= 216.8 Hz, P*C*CO) ppm. **^{7}Li NMR** (156 MHz, THF-d_8) δ = 0.36 ppm. **^{31}P{^{1}H}-NMR** (162 MHz, THF-d_8): δ = 14.03 (s, *P*Ph$_2$O) ppm. **FT-IR** (ATR, cm^{-1}): 2979.3 (bw), 2957.8 (bw), 2833.5 (w), 2109.5 (s, CCO stretching), 1653.0 (w), 1456.9 (w), 1436.2 (s), 1361.5 (m), 1301.2 (m), 1288.9 (m), 1142.5(s), 1114.5 (s), 1094.4 (s),1064.9 (m), 1057.7 (m), 898.3 (w), 789.8 (w), 755.4 (m), 714.5 (s), 695.8 (s), 617.6 (w), 569.4 (w), 529.9 (s), 500.5 (m).

Synthesis of compounds 3b-Li

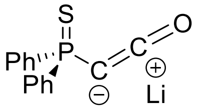

500 mg (1.02 mmol) of **1b** and 123.0 mg (1.12 mmol) of LDA were dissolved in 20 mL tol/thf (9:1) mixture. The solution was stirred for 20 min. After filtration, the atmosphere in the flask was changed from argon to CO and the reaction was stirred for 24 hours. The solution was concentrated to 10 mL. 10 mL pentane were added resulting in the precipitation of a solid. The solid was filtered and washed with toluene (3 times) and pentane (3 times). After drying in vacuo, lithium ketenyl **3b-Li** was obtained as yellowish white solid (241 mg, 0.72 mmol, 71 %).

^{1}H-NMR (400 MHz, THF-*d*$_8$): δ = 8.10 – 7.99 (m, 4H, PPh_2S*H*Ph,*ortho*), 7.24 – 7.17 (m, 6H, PPh_2S*H*Ph,*meta,para*) ppm. **^{13}C{^{1}H}-NMR** (101 MHz, THF-*d*$_8$): δ = 143.8 (d, $^1J_{PC}$ = 95.9 Hz, $PPh_2SeC_{Ph,ipso}$), 138.3 (d, $^2J_{PC}$ = 46.0 Hz, PC*C*O), 131.8 (d, $^2J_{PC}$ = 11.4 Hz, $PPh_2SC_{Ph,ortho}$), 129.6 (d, $^4J_{PC}$ = 3.0 Hz, $PPh_2SC_{Ph,para}$), 127.9 (d, $^3J_{PC}$ = 12.5 Hz, $PPh_2SC_{Ph,meta}$), 6.7 (d, $^1J_{PC}$= 193.8 kHz, P*C*CO) ppm. **^{7}Li NMR** (156 MHz, THF-*d*$_8$) δ = −0.01 ppm. **^{31}P{^{1}H}-NMR** (162 MHz, THF-d$_8$): δ = 21.61 (s, *P*Ph_2S) ppm. **FT-IR** (ATR, cm^{-1}): 3051.1 (bw), 2982.2 (bw), 2878.8 (bw), 2142.0 (s, CCO stretching), 1597.7 (bs), 1571.9 (m), 1477.1 (w), 1435.4 (s), 1404.6 (m), 1100.1 (s), 1069.2 (m), 914.8 (bs), 859.5 (w), 835.1 (w), 743.9 (m), 706.6 (s), 688.6 (s), 675.0 (s), 644.1 (w), 608.9 (m), 550.1 (m), 491.1 (s). Attempts on obtaining satisfactory elemental analysis data were repeatedly unsuccessful.

PMDETA complex of 3b-Li

500 mg (1.02 mmol) of **1b** and 123.0 mg (1.12 mmol) of LDA were dissolved in 20 mL tol/thf (9:1) mixture. The solution was stirred for 20 min. 1.1 eq. of *N,N,N',N"N"*-pentamethyl-diethylenetriamine (PMDETA) (196 mg, 1.12 mmol) added. After filtration, the atmosphere in the flask was changed from argon to CO and the reaction was stirred for 24 hours. The solution was concentrated to 10 mL. 10 mL pentane were added resulting in the precipitation of a solid. The solid was filtered and washed with toluene (3 times) and pentane (3 times). After drying in vacuo, lithium ketenyl **3b-Li**(PMDETA) was obtained as yellowish white solid (302 mg, 0.69 mmol, 68 %).

^{1}H-NMR (400 MHz, THF-*d*$_8$): δ = 8.06 – 7.97 (m, 4H, PPh_2S*H*Ph,*ortho*), 7.24 – 7.19 (m, 6H, PPh_2Se*H*Ph,*meta,para*), 2.43 (t, $^3J_{HH}$ = 6.7 Hz, 4H, C*H*$_2$-PMDETA), 2.33 (t, $^3J_{HH}$ = 6.7 Hz, 4H, C*H*$_2$-PMDETA), 2.24 (s, 3H, C*H*$_3$-PMDETA), 2.17 (s, 12H, C*H*$_3$-PMDETA). **^{13}C{^{1}H}-NMR** (101 MHz, THF-*d*$_8$): δ = 143.7 (d, $^1J_{PC}$ = 94.3 Hz, $PPh_2SC_{Ph,ipso}$), 139.5 (d, $^2J_{PC}$ = 42.0 Hz, PC*C*O), 131.7 (d, $^2J_{PC}$ = 11.5 Hz, $PPh_2SC_{Ph,ortho}$), 129.6 (d, $^4J_{PC}$ = 3.0 Hz, $PPh_2SeC_{Ph,para}$), 127.9 (d, $^3J_{PC}$ = 12.7 Hz, $PPh_2SC_{Ph,meta}$), 58.8 (s, *C*H_2-PMDETA), 56.9 (s, *C*H_2-PMDETA), 46.3 (s, *C*H_3-PMDETA), 43.8 (s, *C*H_3-PMDETA), 5.5 (d, $^1J_{PC}$= 183.2 Hz, P*C*CO) ppm. **^{7}Li NMR** (156 MHz, THF-*d*$_8$) δ = 0.05 ppm. **^{31}P{^{1}H}-NMR** (162 MHz, THF-d$_8$): δ = 22.40 (s, *P*Ph_2S) ppm. **FT-IR** (ATR, cm^{-1}): 3051.1 (bw), 2957.8 (bw), 2829.9 (bw), 2789.7 (bw), 2103.9 (s, CCO stretching), 1621.4 (m), 1542.4 (w), 1456.9 (m), 1435.4 (s), 1361.5 (m), 1352.1 (m), 1290.4 (m), 1253.05 (m), 1167.6 (w), 743.93 (m), 690.8 (s), 638.4 (m), 561.5 (m), 499.07 (s).

Synthesis of compounds 3c-Li

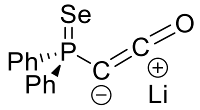

200 mg (0.37 mmol) of **1c** and 45.1 mg (0.41 mmol) of LDA were dissolved in 10 mL tol/thf(9:1) mixture. The solution was stirred for 20 min. After filtration the atmosphere in the flask was changed from argon to CO and the reaction was stirred for 24 hours. The solution was concentrated to 5 mL. Subsequently, 5 mL pentane were added resulting in the precipitation of a solid. The solid was filtered and washed with toluene (3 times) and hexane (3 times). After drying in vacuo, lithium ketenyl **3c-Li** was obtained as off-white solid (82 mg, 0.37 mmol, 58 %). **3c-Li** is not stable for longer time in THF solution, but slowly converts to benzene derivative **4**.

^{1}H-NMR (400 MHz, THF-*d*$_8$): δ = 8.10 – 7.99 (m, 4H, PPh_2Se*H*Ph,*ortho*), 7.25 – 7.17 (m, 6H, PPh_2Se*H*Ph,*meta,para*), 3.65 – 3.59 (m, 2H, THF-OC*H*$_2$), 1.83 – 1.74 (m, 2H, THF-OCH$_2$C*H*$_2$). **^{13}C{^{1}H}-NMR** (101 MHz, THF-*d*$_8$): δ = 142.7 (d, $^1J_{PC}$ = 87.3 Hz, $PPh_2SeC_{Ph,ipso}$), 139.8 (d, $^2J_{PC}$ = 45.0 Hz, PC*C*O), 132.1 (d, $^2J_{PC}$ = 11.8 Hz, $PPh_2SeC_{Ph,ortho}$), 129.9 (d, $^4J_{PC}$ = 3.0 Hz, $PPh_2SeC_{Ph,para}$), 127.9 (d, $^3J_{PC}$ = 12.7 Hz, $PPh_2SeC_{Ph,meta}$), 68.4 (s, THF-O*C*H$_2$CH$_2$), 26.5 (s, THF-OCH$_2$*C*H$_2$), 5.9 (d, $^1J_{PC}$= 184.9 Hz, P*C*CO) ppm. **^{7}Li NMR** (156 MHz, THF-*d*$_8$) δ = –0.13. **^{31}P{^{1}H}-NMR** (162 MHz, THF-d$_8$): δ = 5.57 (s, *P*Ph$_2$Se) ppm. **^{77}Se-NMR** (76 MHz, THF-*d*$_8$): δ = 143.17 (d, $^1J_{PSe}$ = 703.1 Hz, PPh$_2$*Se*) ppm. **FT-IR** (ATR, cm^{-1}): 3051.8 (bw), 2969.3 (bw), 2875.19 (bw), 2138.4 (s, CCO stretching), 1749.2 (bs), 1541.4 (w), 1435.4 (s), 1403.1 (w), 1307.6 (s), 1096.5 (s), 1043.4 (m), 1027.6(m), 998.1 (m), 915.5 (w), 742.5 (m), 688.6 (s), 646.3 (m), 529.2 (s), 486.9 (s). **Anal. Calcd.** for $C_{18}H_{18}Li_1O_2P_1Se_1$: C, 56.42; H, 4.73. Found: C, 56.80; H, 4.94.

PMDETA complex of 3c-Li

200 mg (0.37 mmol) of **1c** and 45.1 mg (0.41 mmol) of LDA were dissolved in 10 mL tol/thf(9:1) mixture. The solution was stirred for 20 min. 1.1 eq. of *N,N,N',N"N"*-pentamethyl-diethylenetriamine (PMDETA) (71.5 mg, 0.41 mmol) was added. After filtration the atmosphere in the flask was changed from argon to CO and the reaction was stirred for 24 hours. The solution was concentrated to 5 mL. Subsequently, 5 mL pentane were added resulting in the precipitation of a solid. The solid was filtered and washed with toluene (3 times) and hexane (3 times). After drying in vacuo, lithium ketenyl **3c-Li**(PMDETA) was obtained as off-white solids off-white solid (129 mg, 0.27 mmol, 72 %). Single crystal of **3c-Li**(PMDETA) were grown by slow diffusion of hexane into a toluene/THF solution of the compound at –30 °C.

^{1}H-NMR (400 MHz, THF-*d*$_8$): δ = 8.08 – 8.00 (m, 4H, PPh_2Se*H*Ph,*ortho*), 7.25 – 7.16 (m, 6H, PPh_2Se*H*Ph,*meta,para*), 2.44 (t, $^3J_{HH}$ = 6.7 Hz, 4H, C*H*$_2$-PMDETA), 2.33 (t, $^3J_{HH}$ = 6.7 Hz, 4H, C*H*$_2$-PMDETA), 2.24 (s, 3H, C*H*$_3$-PMDETA), 2.18 (s, 12H, C*H*$_3$-PMDETA). **^{13}C{^{1}H}-NMR** (101 MHz, THF-*d*$_8$): δ = 142.7 (d, $^1J_{PC}$ = 86.5 Hz, $PPh_2SeC_{Ph,ipso}$), 140.5 (d, $^2J_{PC}$ = 43.0 Hz, PC*C*O), 132.0 (d, $^2J_{PC}$ = 11.9 Hz, $PPh_2SeC_{Ph,ortho}$), 129.7 (d, $^4J_{PC}$ = 3.0 Hz, $PPh_2SeC_{Ph,para}$), 127.9 (d, $^3J_{PC}$ = 12.6 Hz, $PPh_2SeC_{Ph,meta}$), 58.8 (s, *C*H$_2$-PMDETA), 56.8 (s, *C*H$_2$-PMDETA), 46.3 (s, *C*H$_3$-PMDETA), 43.9 (s, *C*H$_3$-PMDETA), 5.5 (d, $^1J_{PC}$= 178.4 Hz, P*C*CO) ppm. . **^{7}Li NMR** (156 MHz, THF-*d*$_8$) δ = –0.11 ppm. **^{31}P{^{1}H}-NMR** (162 MHz, THF-d$_8$): δ = 6.17 (s, *P*Ph$_2$Se) ppm. **^{77}Se-NMR** (76 MHz, THF-*d*$_8$): δ = 146.55 (d, $^1J_{PSe}$ = 694.7 Hz, PPh$_2$*Se*) ppm. **FT-IR** (ATR, cm^{-1}): 3047.5 (bw), 2952.7 (bw), 2855.1 (bw), 2829.2 (bw), 2786.2 (bw), 2098.2 (s, CCO stretching),

1604.2 (bw), 1470.6 (m), 1457.7 (m), 1436.1 (s), 1304.03 (w), 1094.4 (s), 1062.7 (m), 1019.7 (s), 936.7 (m), 705.2 (m), 690.8 (m), 667.8 (m), 529.9 (s), 492.6 (s), 472.5 (w), 429.4 (m).

2. NMR and IR spectra

2.1. NMR spectra of the metallated ylides 2

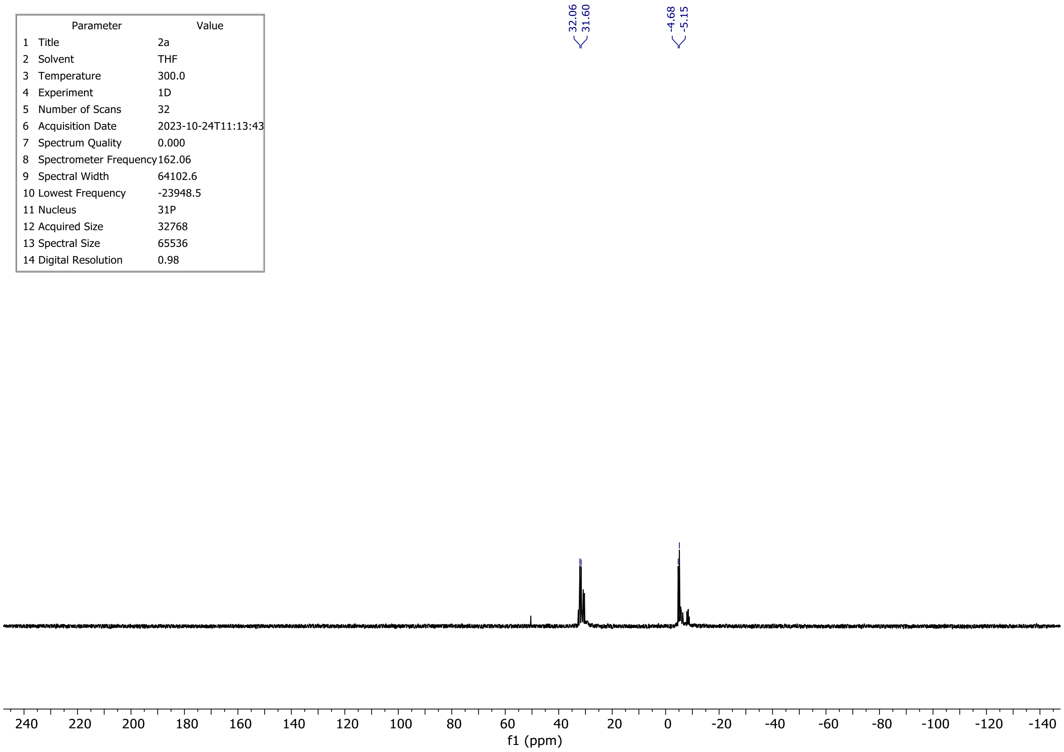

Figure S1. $^{31}P\{^{1}H\}$ NMR spectrum of compound **2a** in THF-d_8.

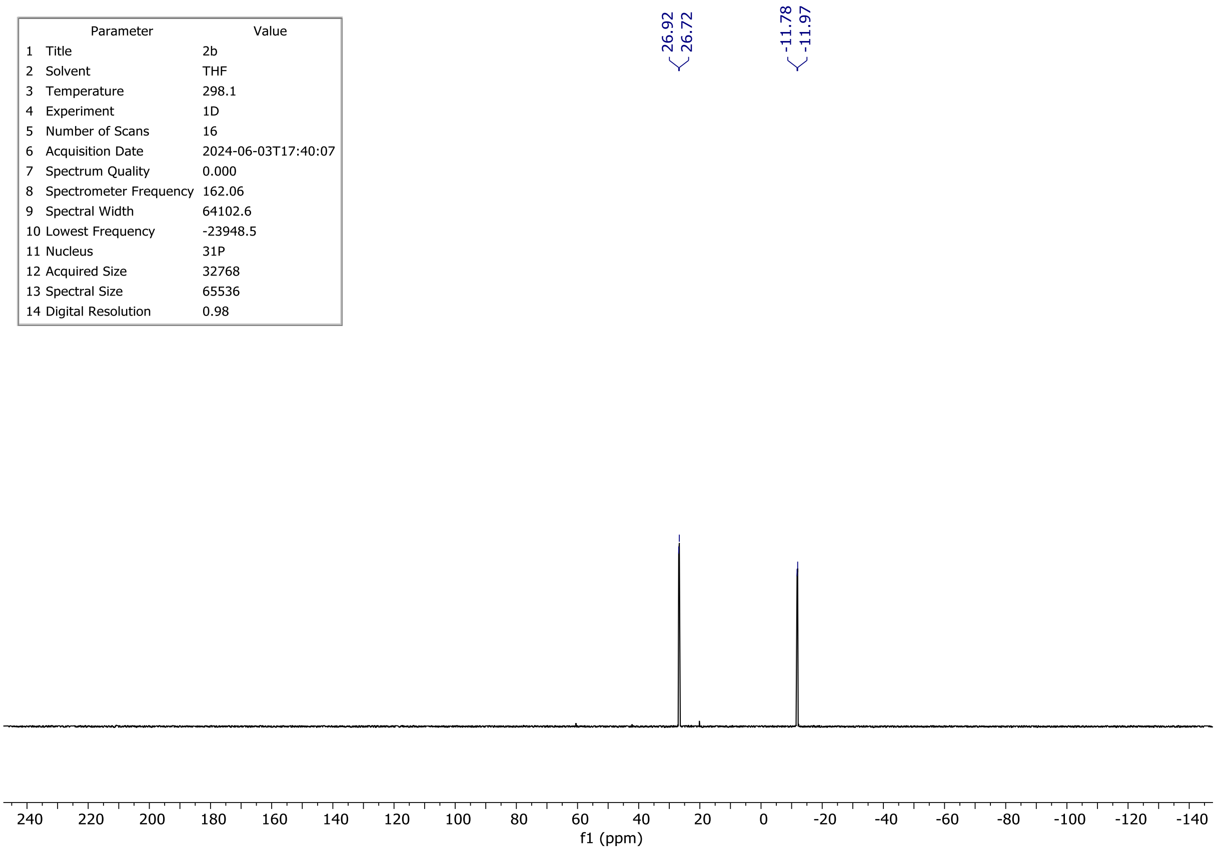

Figure S2. $^{31}P\{^{1}H\}$ NMR spectrum of compound **2b** in THF-d_8.

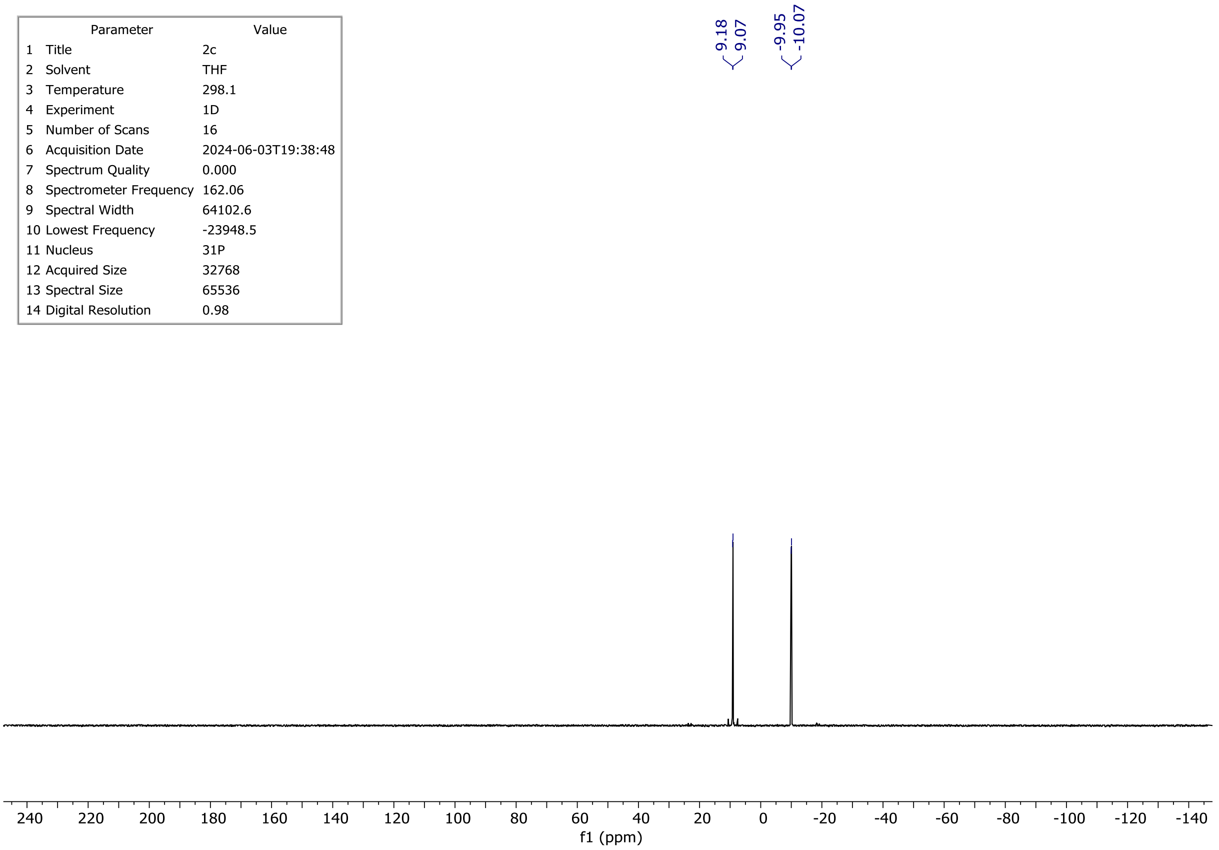

Figure S3. $^{31}P\{^{1}H\}$ NMR spectrum of compound **2c** in THF-d_8.

2.2. NMR and IR spectra of compound 3a-Li

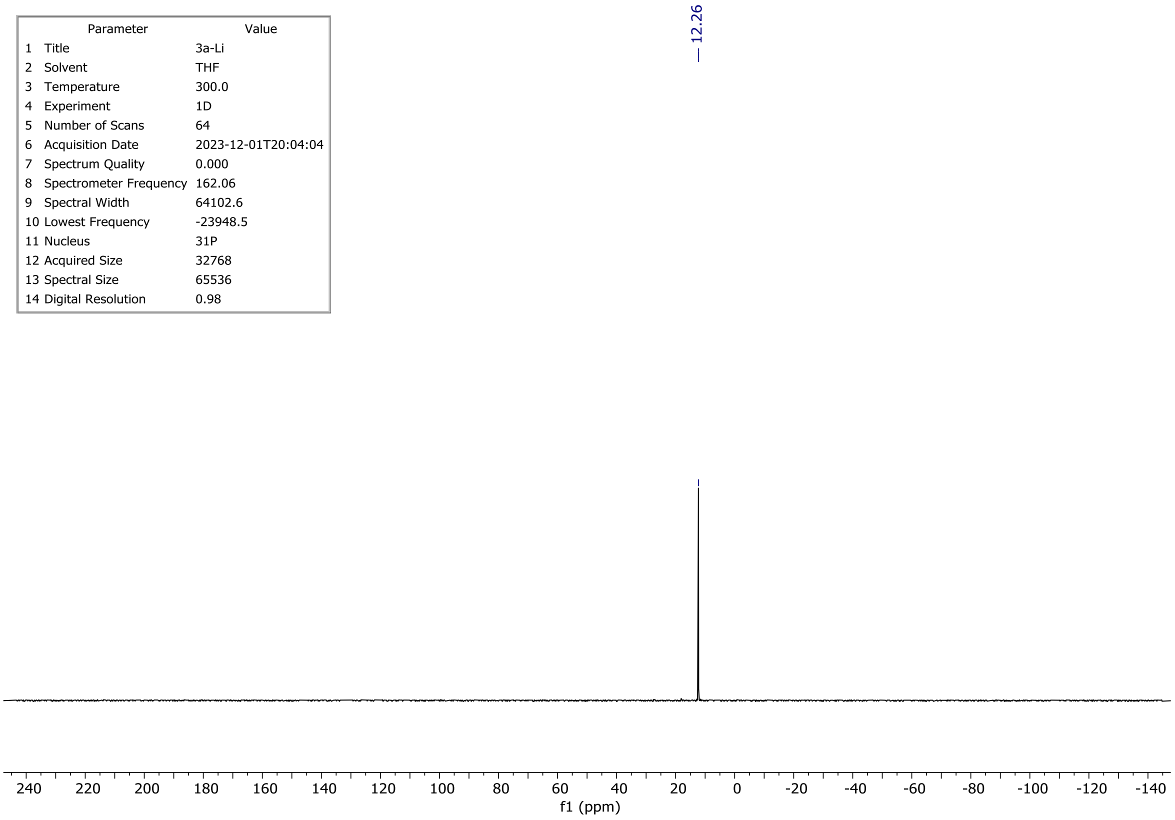

Figure S4. $^{31}P\{^{1}H\}$ NMR spectrum of compound **3a-Li** in THF-d_8.

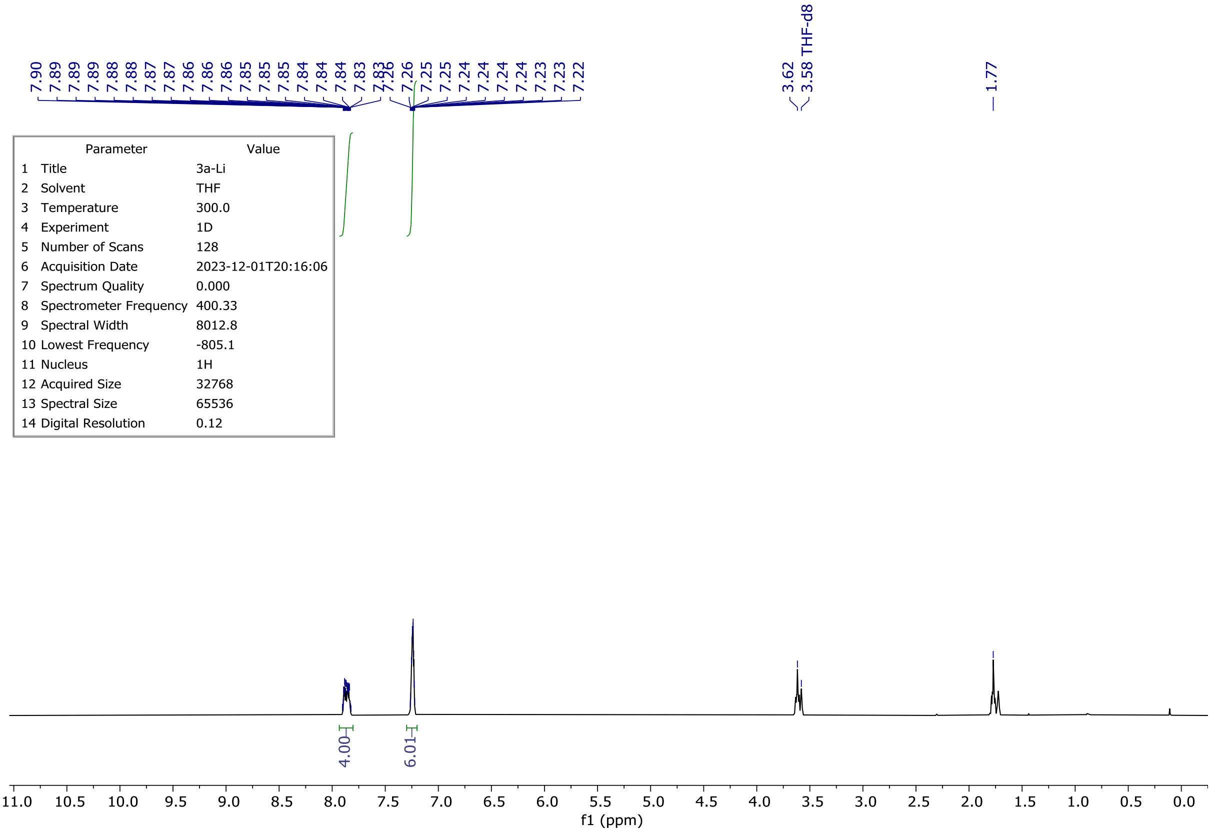

Figure S5. ^{1}H NMR spectrum of compound **3a-Li** in THF-d_8.

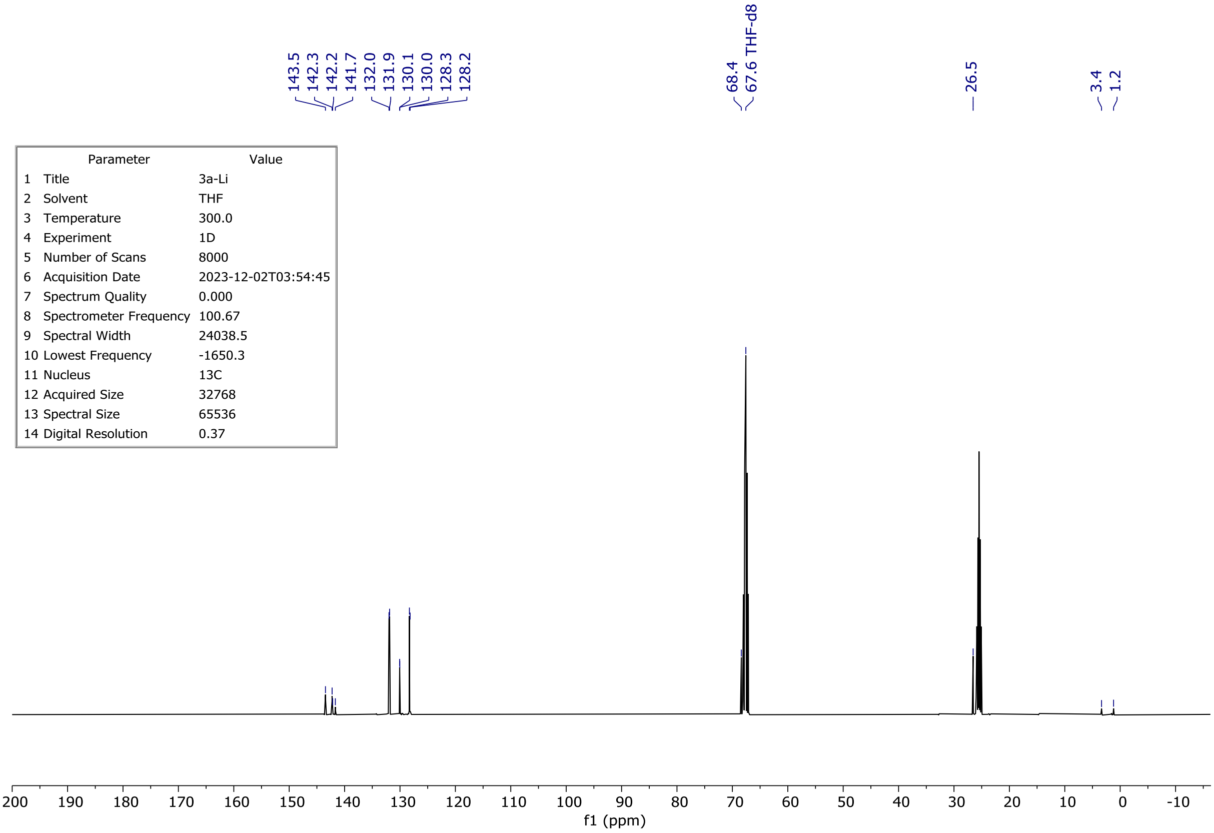

Figure S6. ^{13}C{^{1}H} NMR spectrum of compound **3a-Li** in THF-d_8.

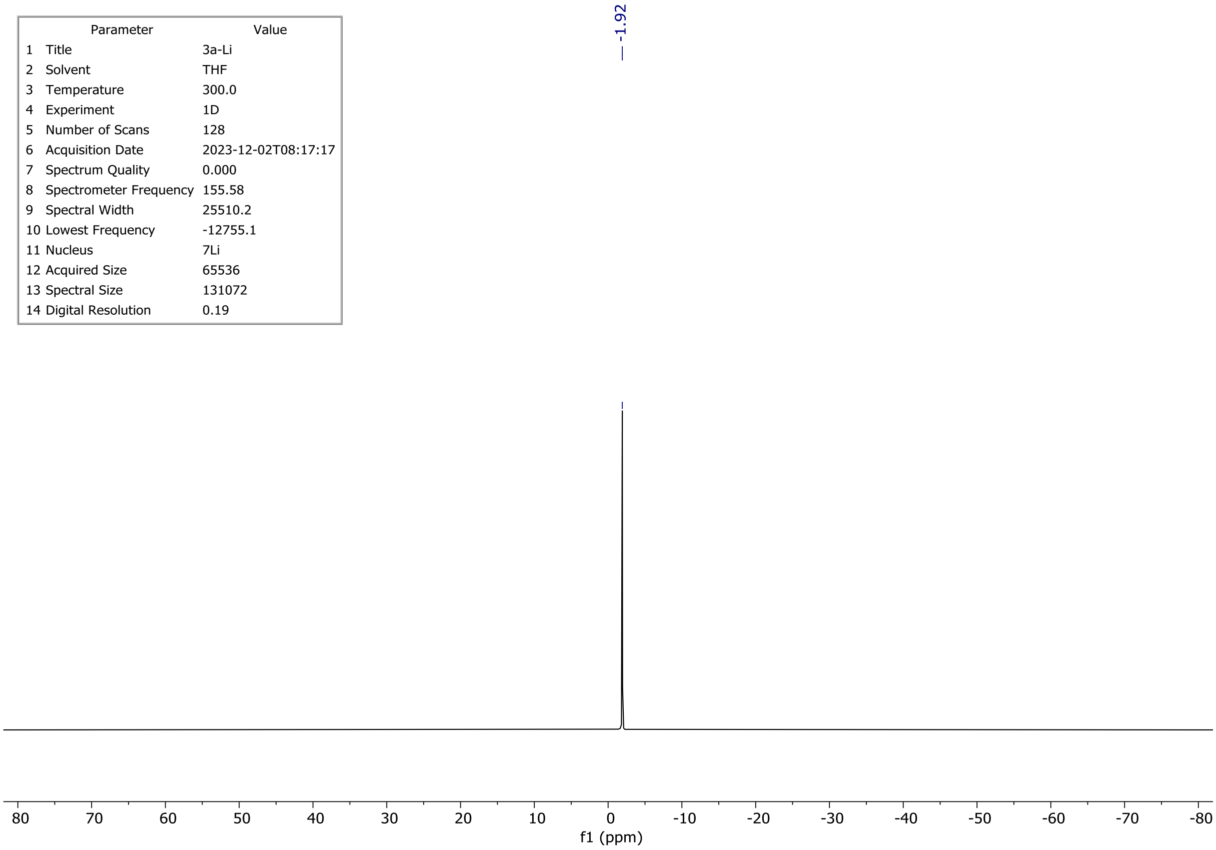

Figure S7. ^{7}Li NMR spectrum of compound **3a-Li** in THF-d_8.

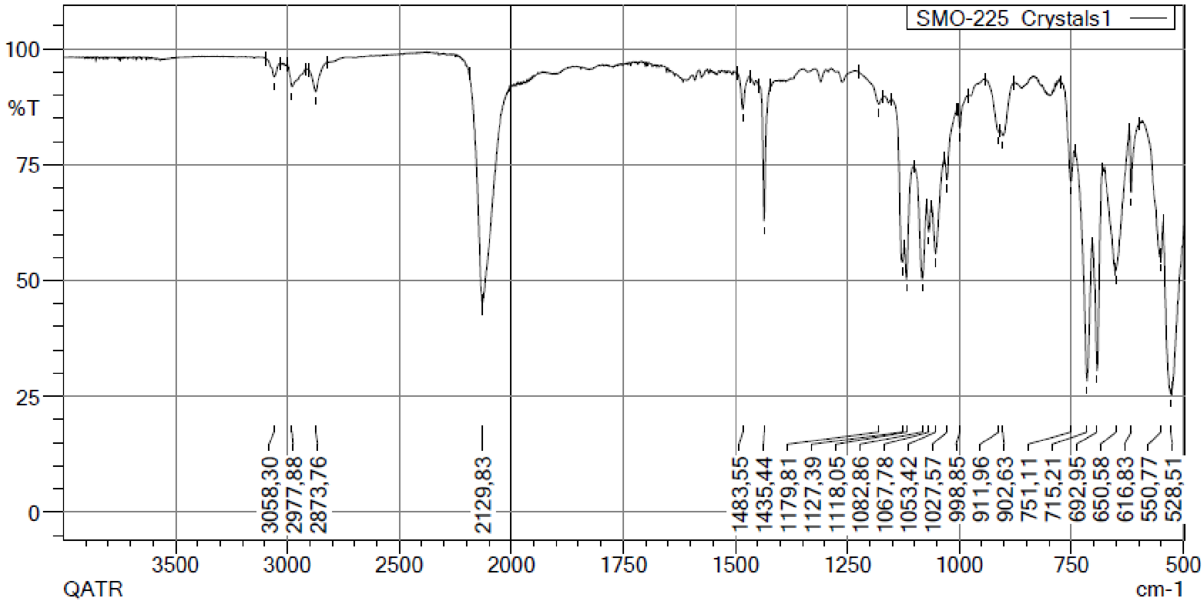

Figure S8. IR spectrum of compound **3a-Li** (solid state).

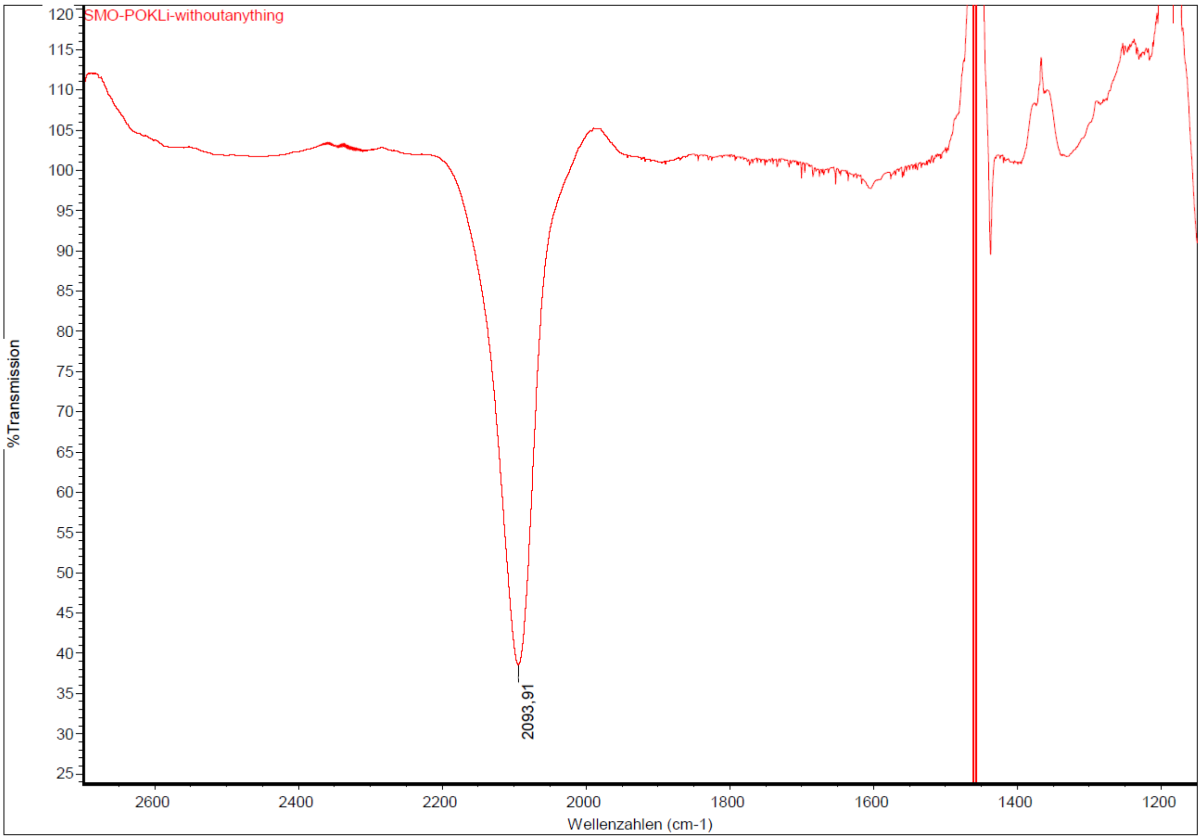

Figure S9. IR spectrum of compound **3a-Li** (THF solution).

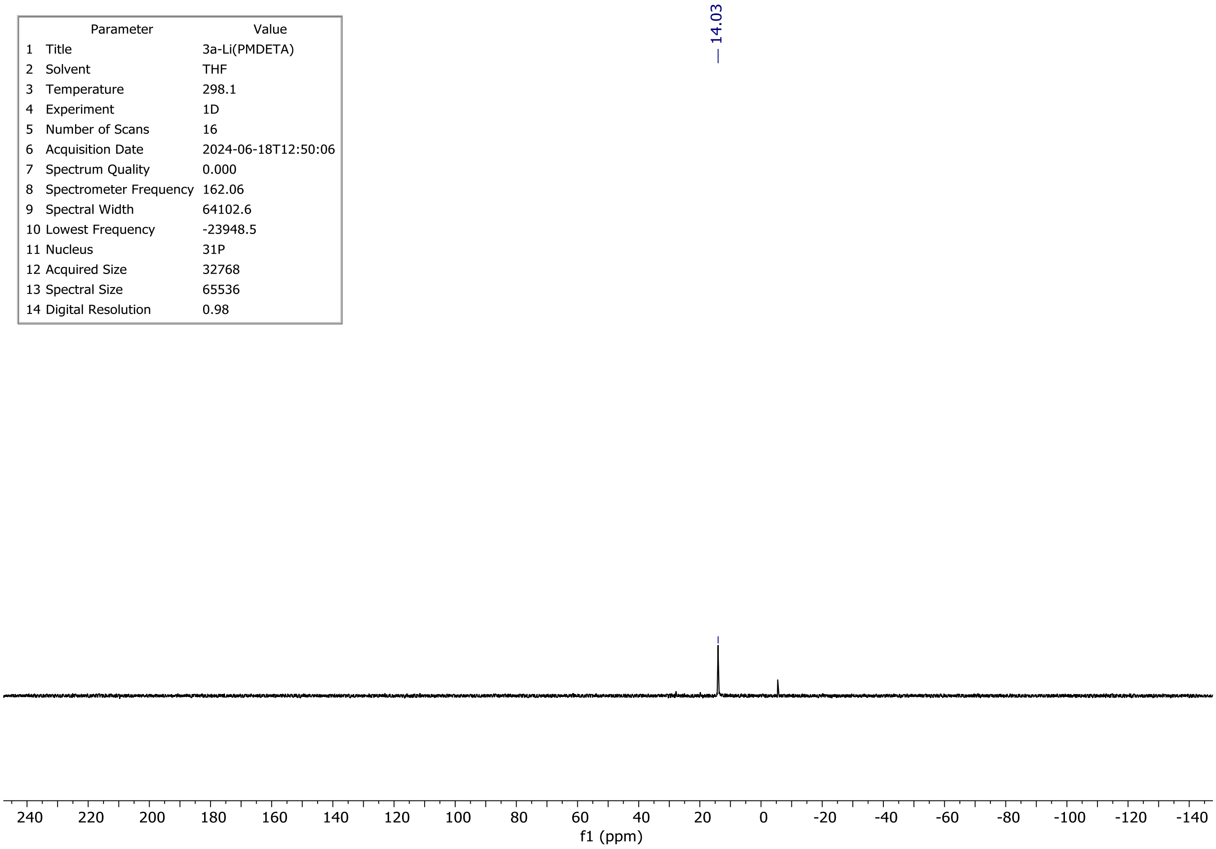

Figure S10. $^{31}P\{^{1}H\}$ NMR spectrum of compound **3a-Li**(PMDETA) in THF-d8.

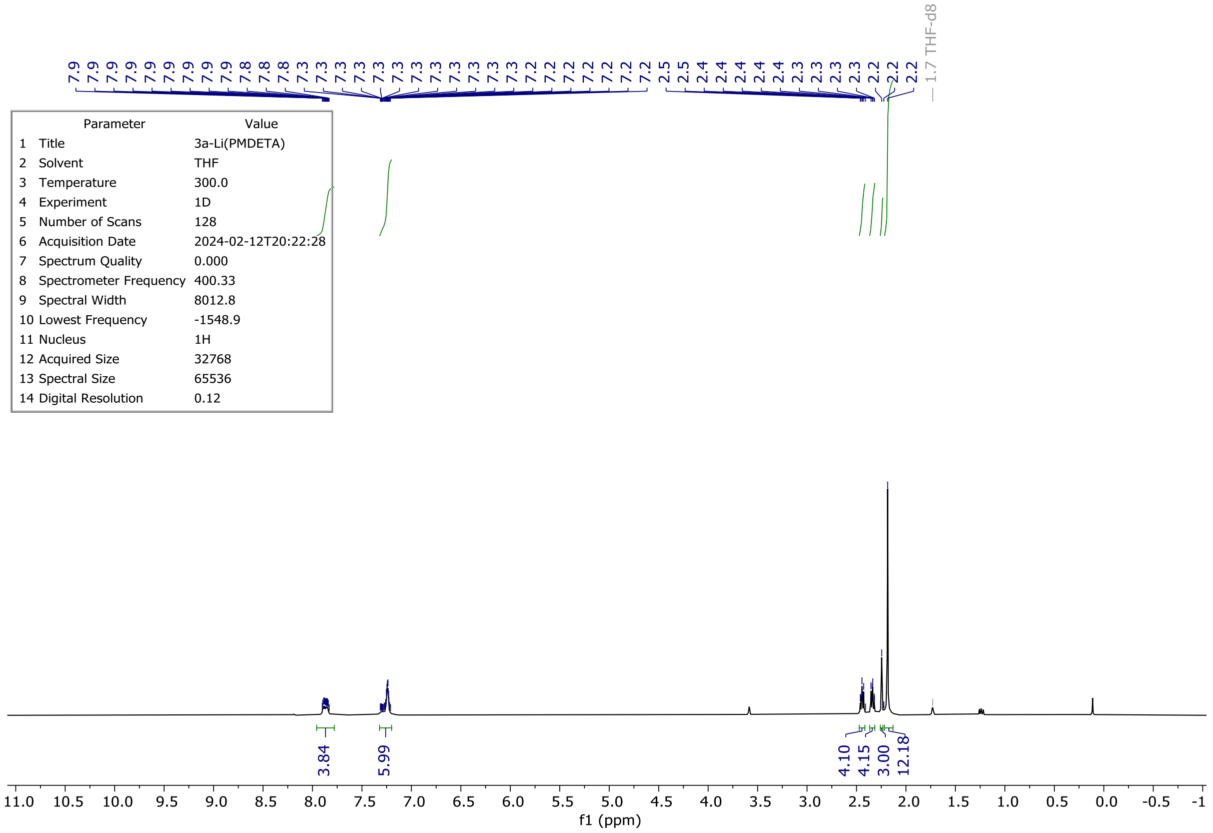

Figure S11. ^{1}H NMR spectrum of compound **3a-Li**(PMDETA) in THF-d_8.

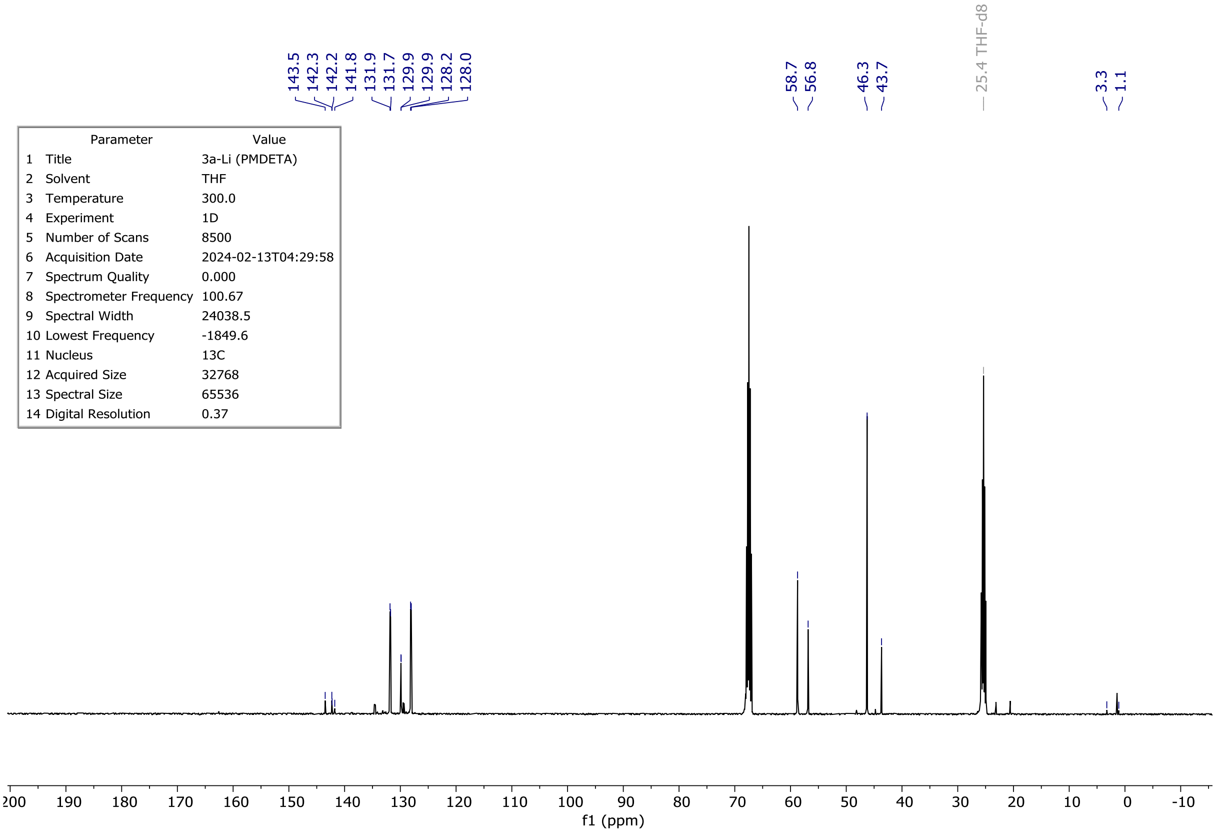

Figure S12. ^{13}C{^{1}H} NMR spectrum of compound **3a-Li**(PMDETA) in THF-d_8.

	Parameter	Value
1	Title	3a-Li(PMDETA)
2	Solvent	THF
3	Temperature	298.0
4	Experiment	1D
5	Number of Scans	32
6	Acquisition Date	2024-06-18T12:55:36
7	Spectrum Quality	0.000
8	Spectrometer Frequency	155.58
9	Spectral Width	25510.2
10	Lowest Frequency	-12755.1
11	Nucleus	7Li
12	Acquired Size	65536
13	Spectral Size	131072
14	Digital Resolution	0.19

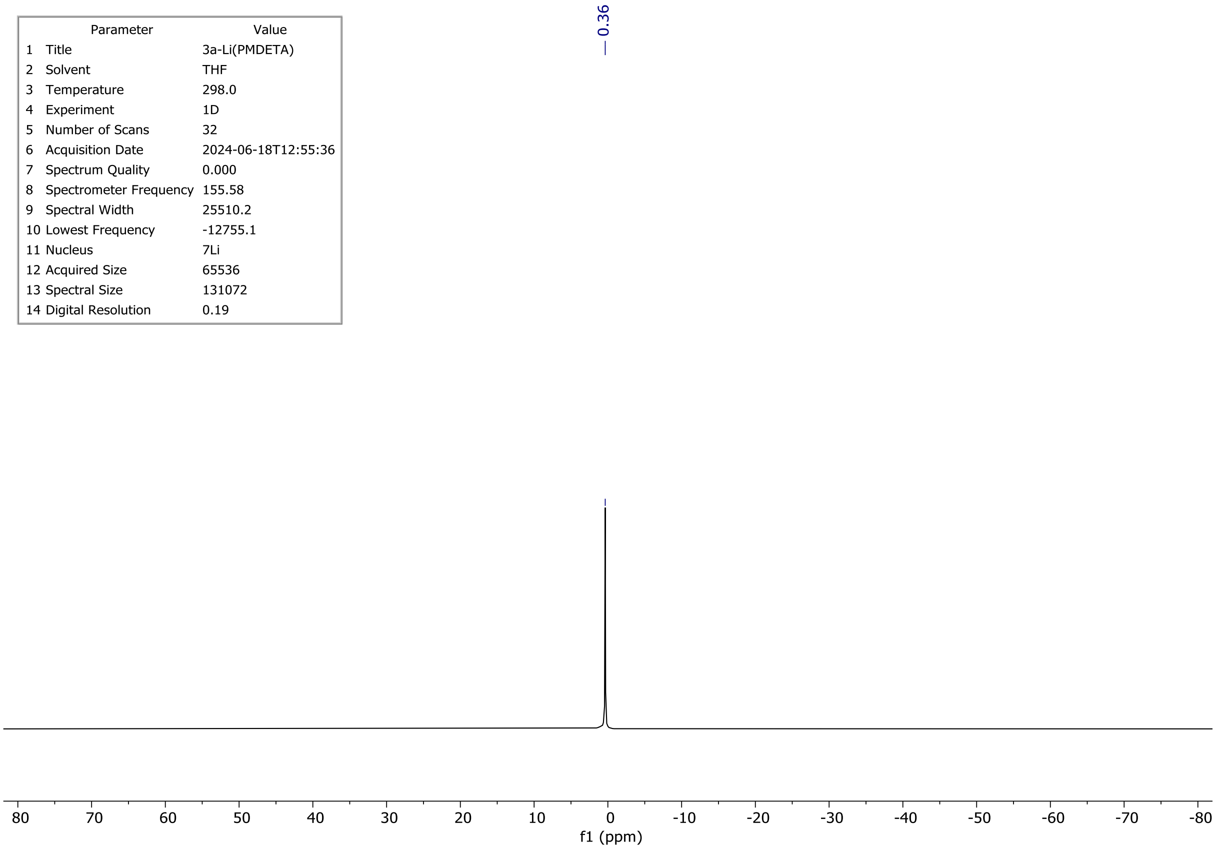

Figure S13. ^{7}Li NMR spectrum of compound **3a-Li**(PMDETA) in THF-d_8.

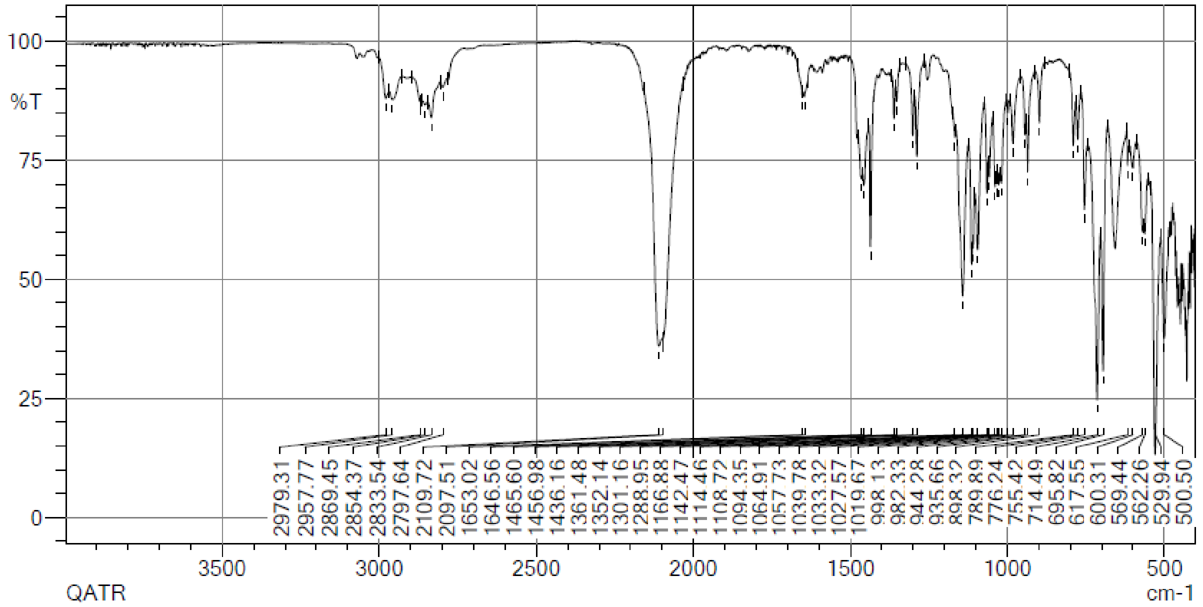

Figure S14. IR spectrum of compound **3a-Li**(PMDETA) (solid state).

2.3. NMR and IR spectra of compound 3b-Li

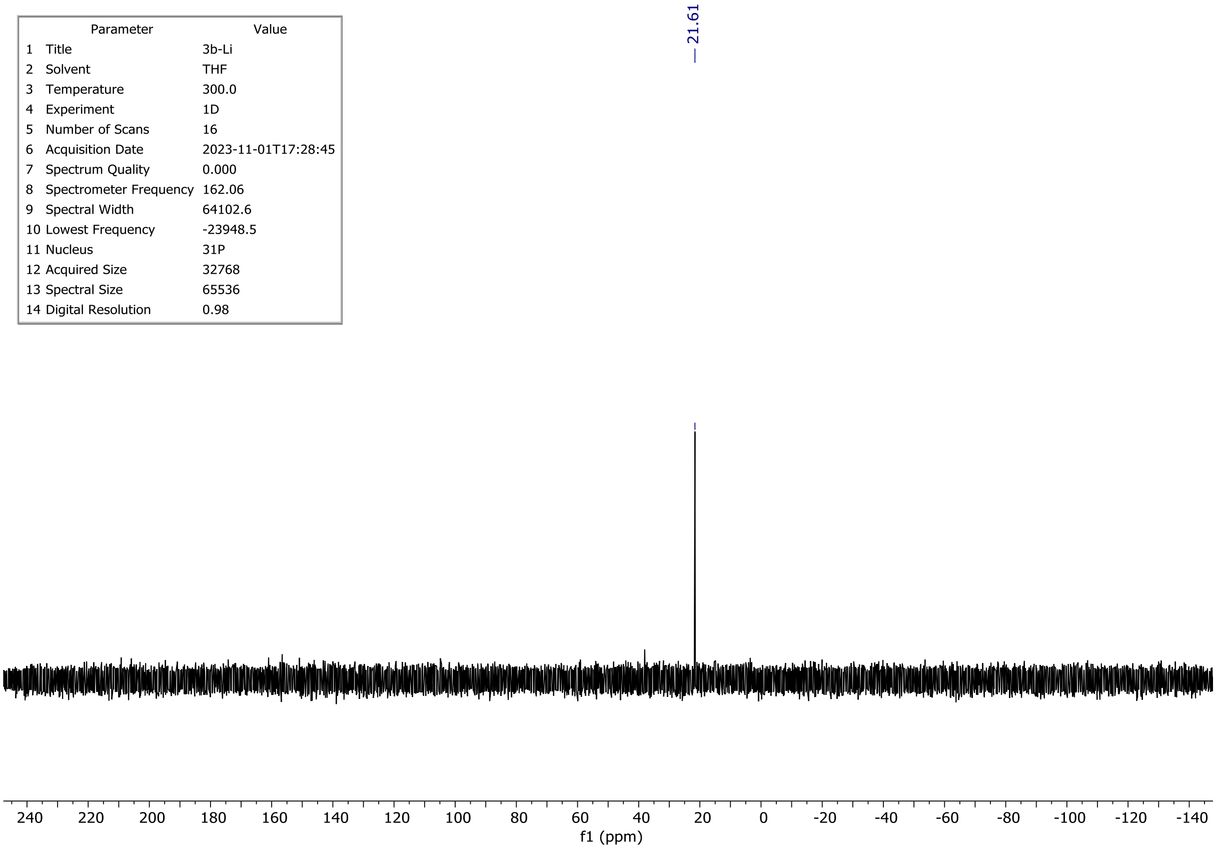

	Parameter	Value
1	Title	3b-Li
2	Solvent	THF
3	Temperature	300.0
4	Experiment	1D
5	Number of Scans	16
6	Acquisition Date	2023-11-01T17:28:45
7	Spectrum Quality	0.000
8	Spectrometer Frequency	162.06
9	Spectral Width	64102.6
10	Lowest Frequency	-23948.5
11	Nucleus	31P
12	Acquired Size	32768
13	Spectral Size	65536
14	Digital Resolution	0.98

Figure S15. $^{31}P\{^{1}H\}$ NMR spectrum of compound **3b-Li** in THF-d_8.

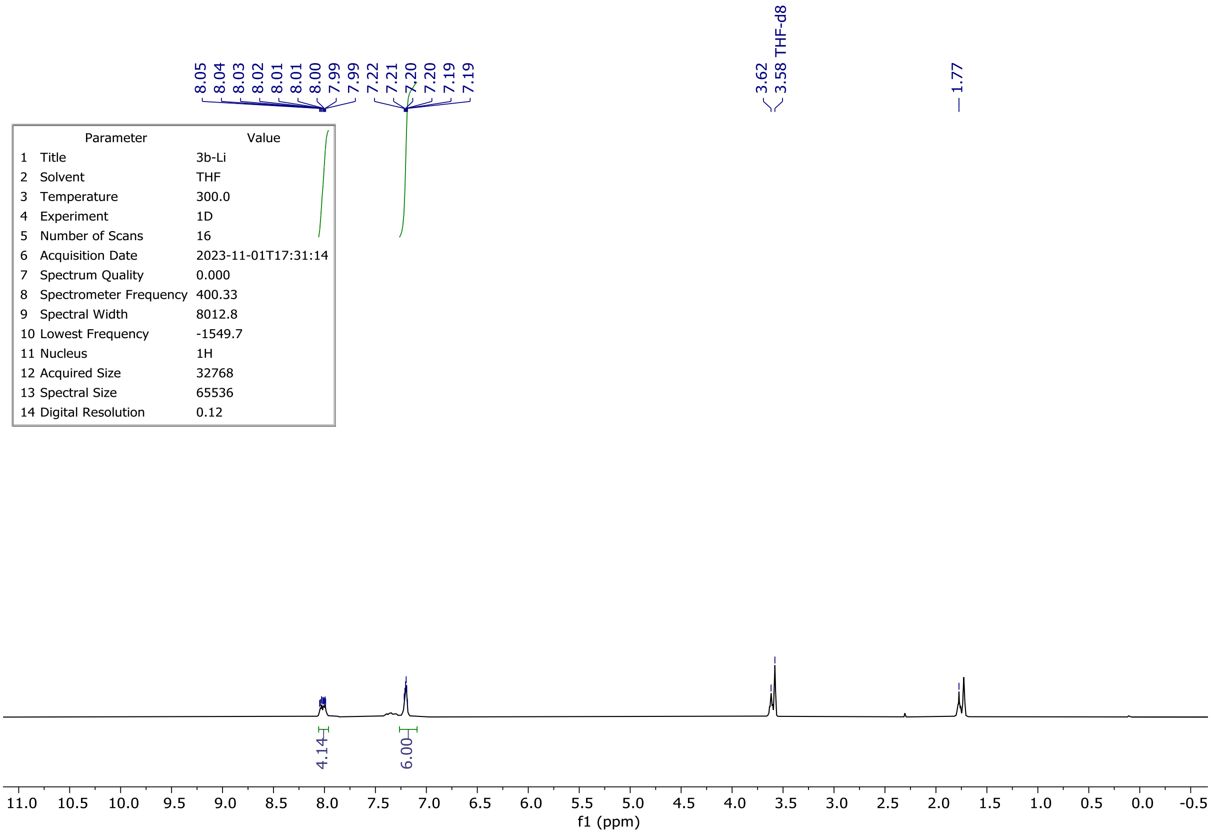

	Parameter	Value
1	Title	3b-Li
2	Solvent	THF
3	Temperature	300.0
4	Experiment	1D
5	Number of Scans	16
6	Acquisition Date	2023-11-01T17:31:14
7	Spectrum Quality	0.000
8	Spectrometer Frequency	400.33
9	Spectral Width	8012.8
10	Lowest Frequency	-1549.7
11	Nucleus	1H
12	Acquired Size	32768
13	Spectral Size	65536
14	Digital Resolution	0.12

Figure S16. ^{1}H NMR spectrum of compound **3b-Li** in THF-d_8.

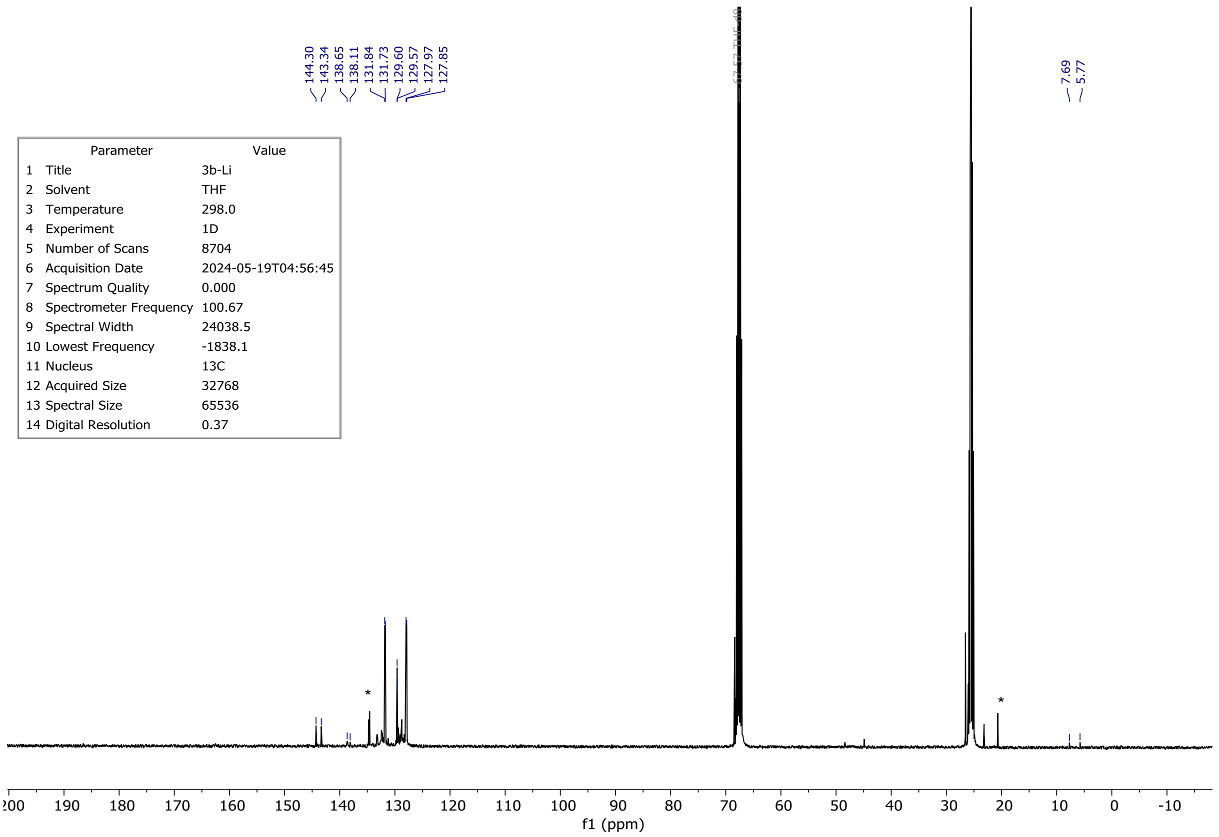

Figure S17. $^{13}C\{^{1}H\}$ NMR spectrum of compound **3b-Li** in THF-d_8.

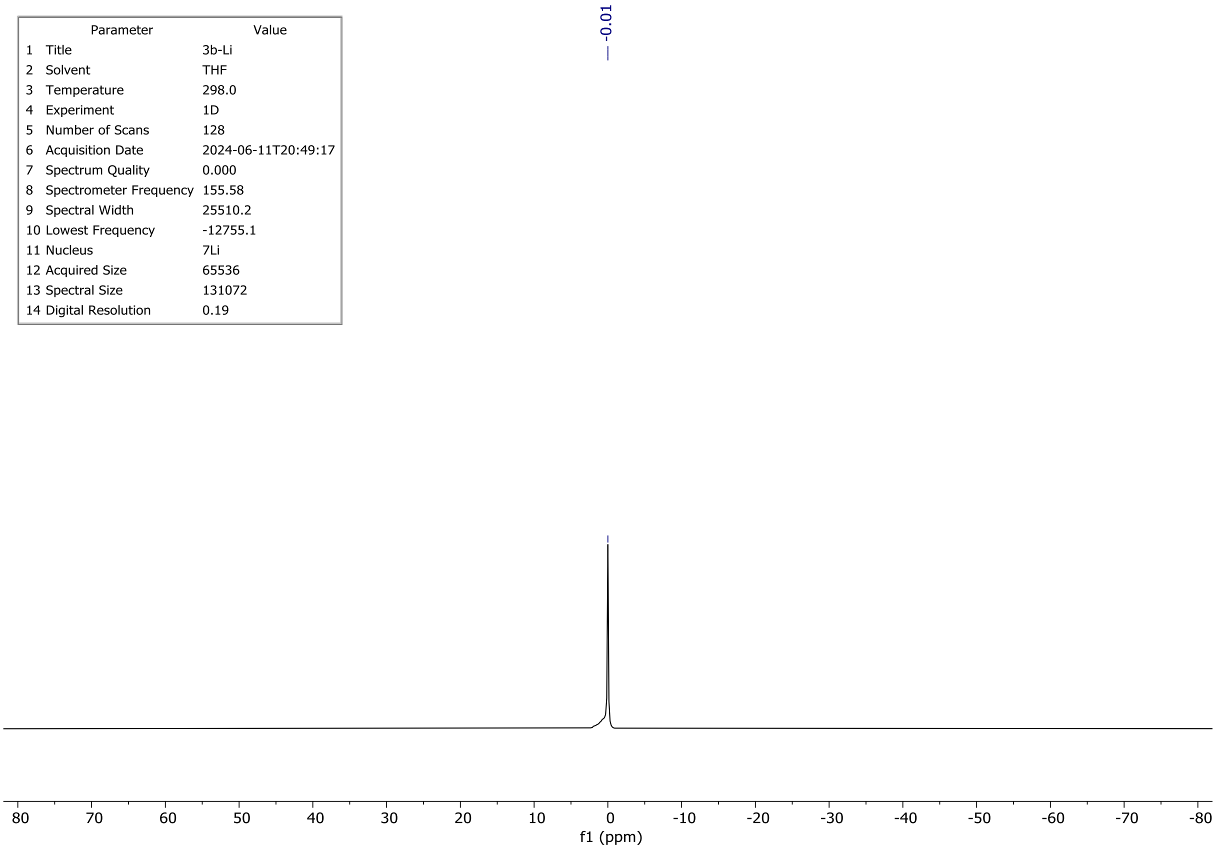

Figure S18. ^{7}Li NMR spectrum of compound **3b-Li** in THF-d_8.

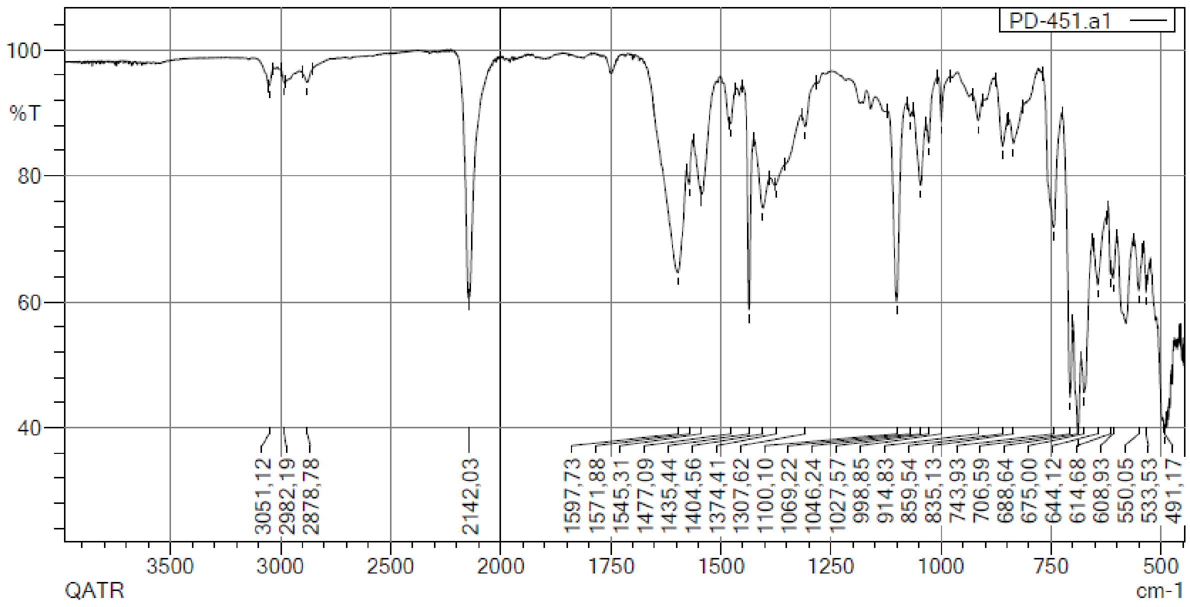

Figure S19. IR spectrum of compound **3b-Li** (solid state).

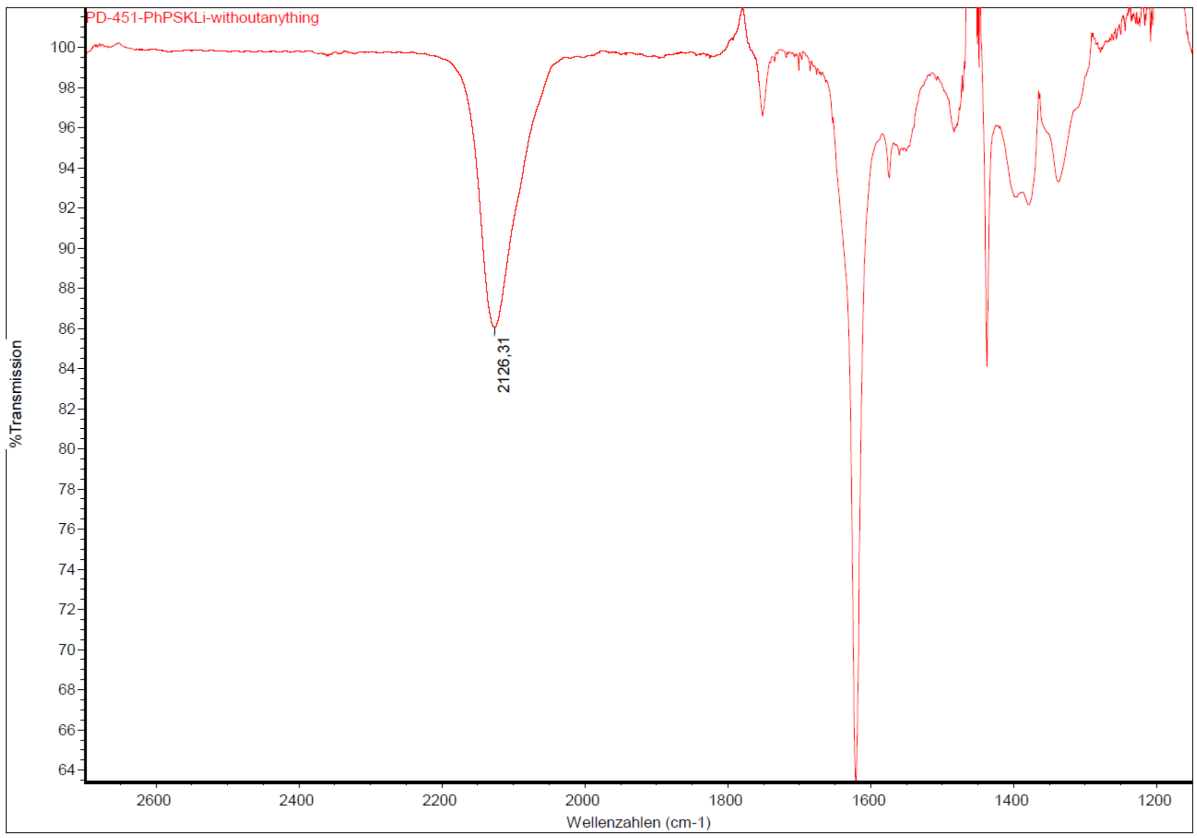

Figure S20. IR spectrum of compound **3b-Li** (THF solution).

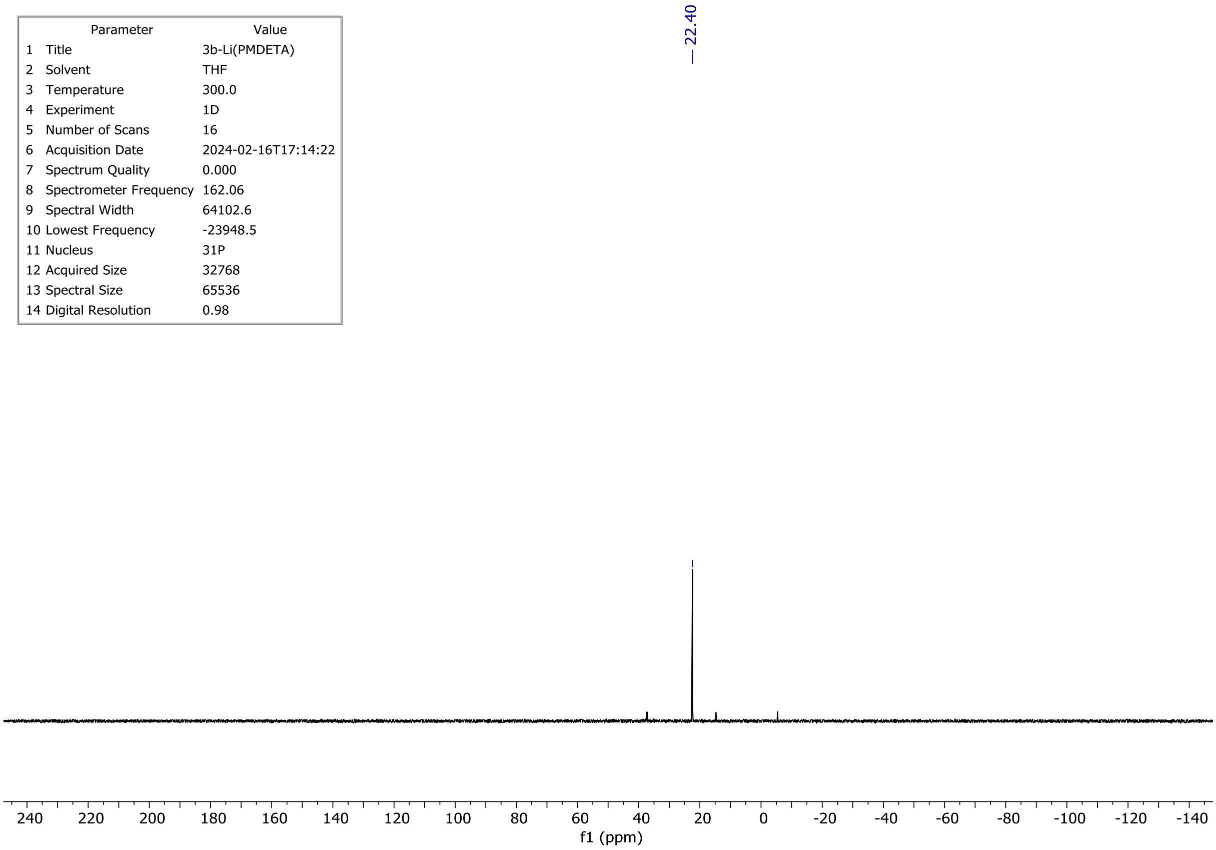

Figure S21. $^{31}P\{^{1}H\}$ NMR spectrum of compound **3b-Li**(PMDETA) in THF-d_8.

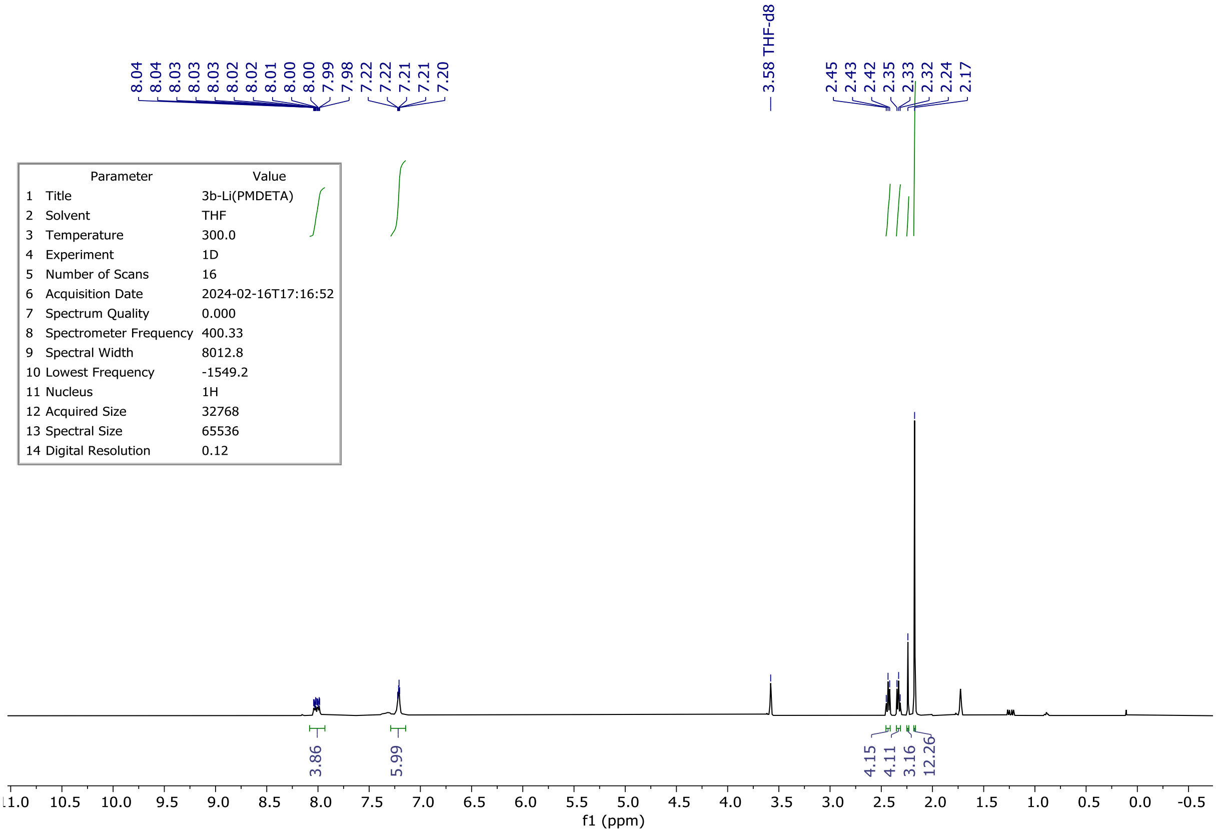

Figure S22. ^{1}H NMR spectrum of compound **3b-Li**(PMDETA) in THF-d_8.

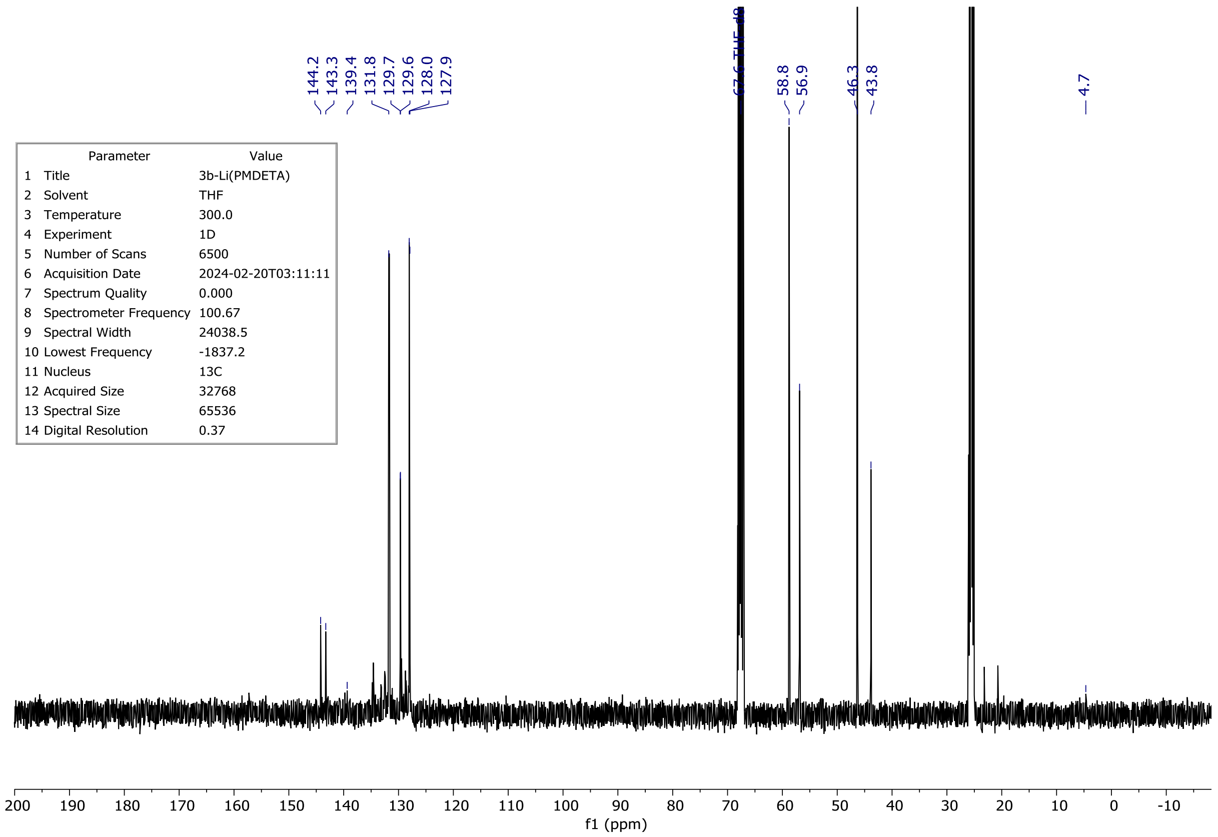

Figure S23. $^{13}C\{^{1}H\}$ NMR spectrum of compound **3b-Li**(PMDETA) in THF-d_8.

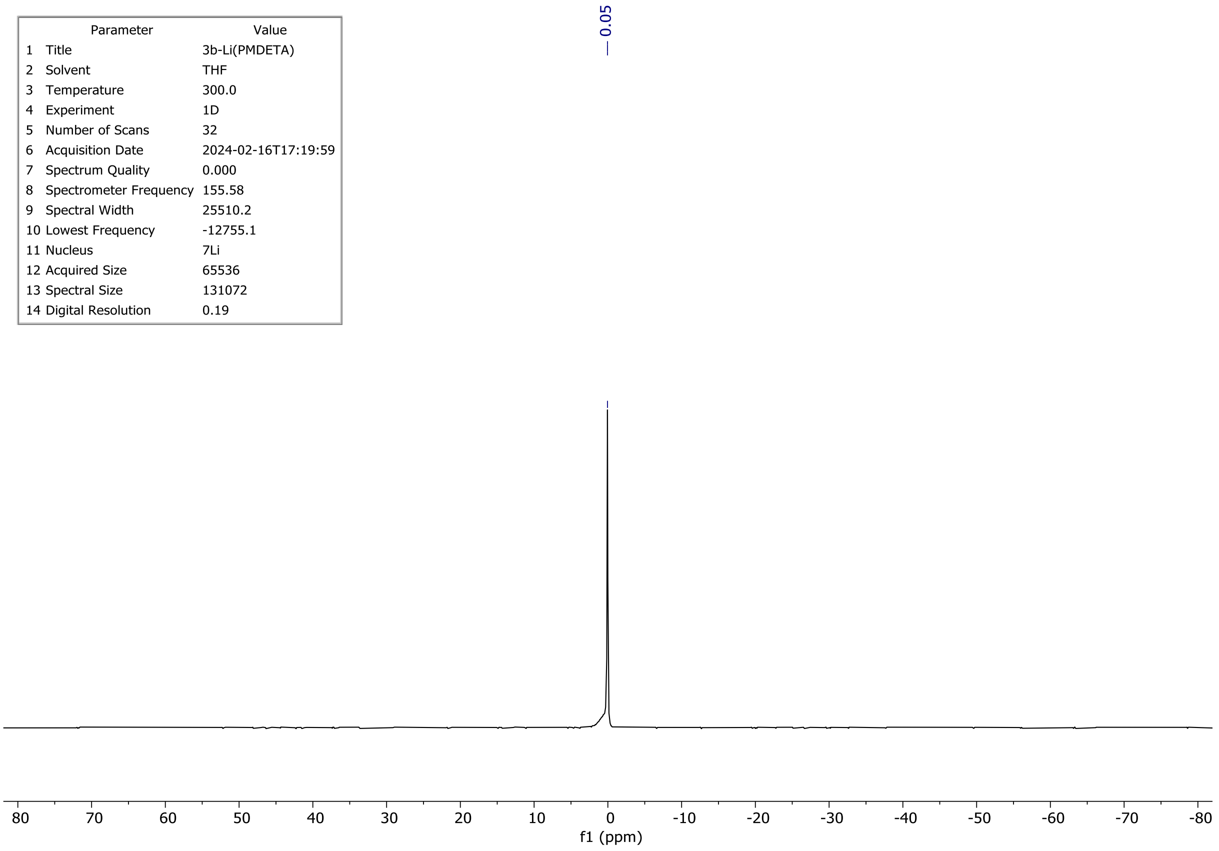

Figure S24. ^{7}Li NMR spectrum of compound **3b-Li**(PMDETA) in THF-d_8.

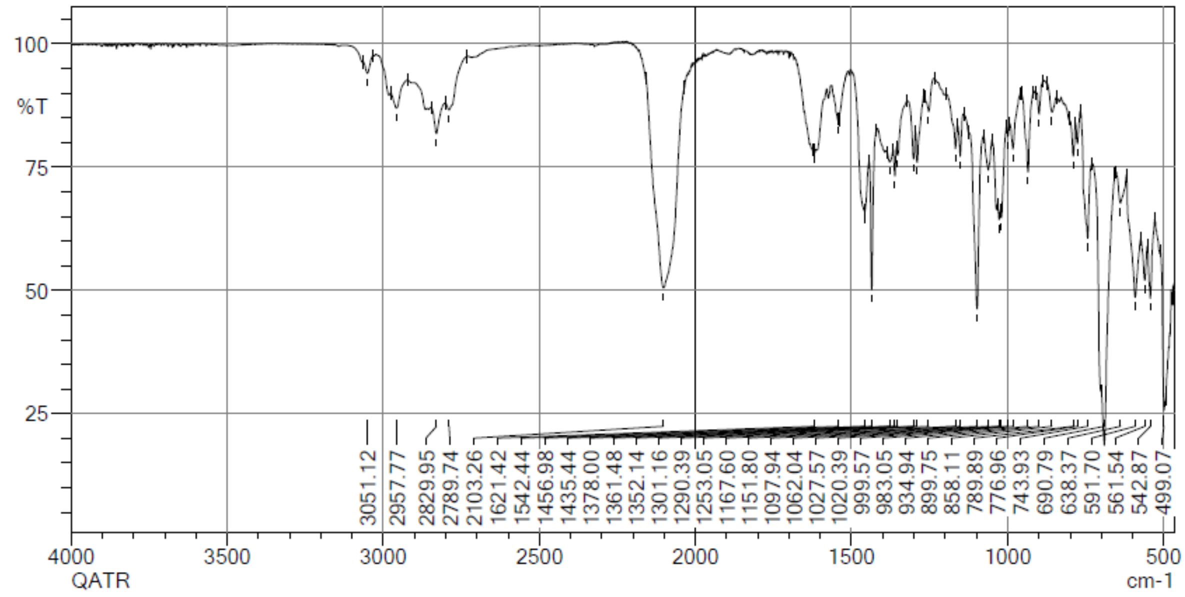

Figure S25. IR spectrum of compound **3b-Li**(PMDETA) (solid state).

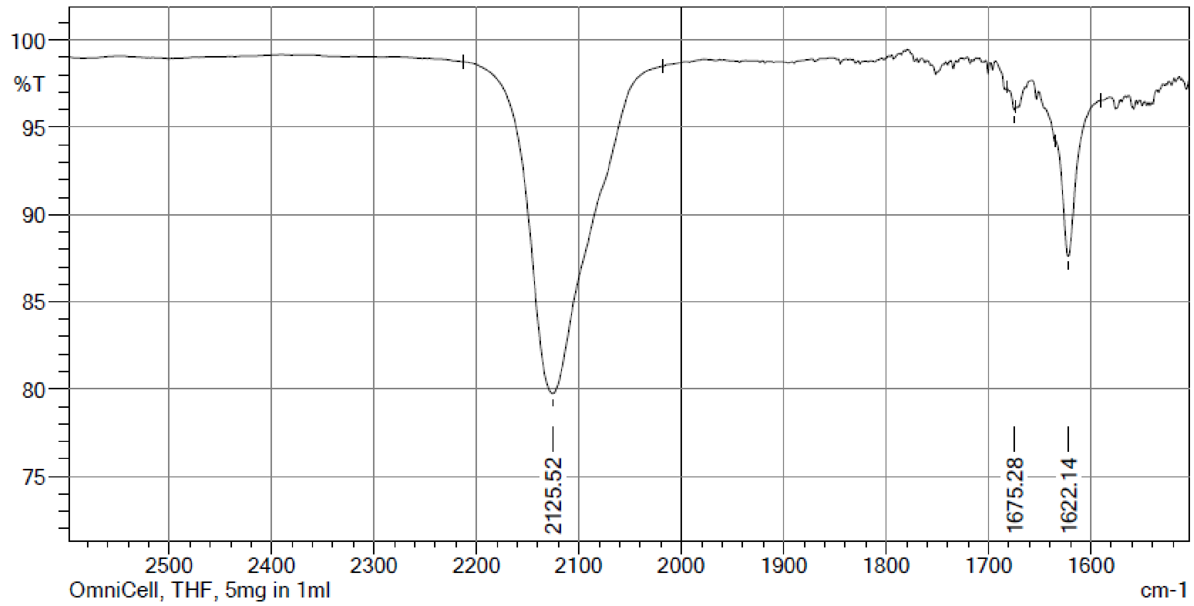

Figure S26. IR spectrum of compound **3b-Li**(PMDETA) (THF solution).

2.4. NMR and IR Spectra of compound 3c-Li

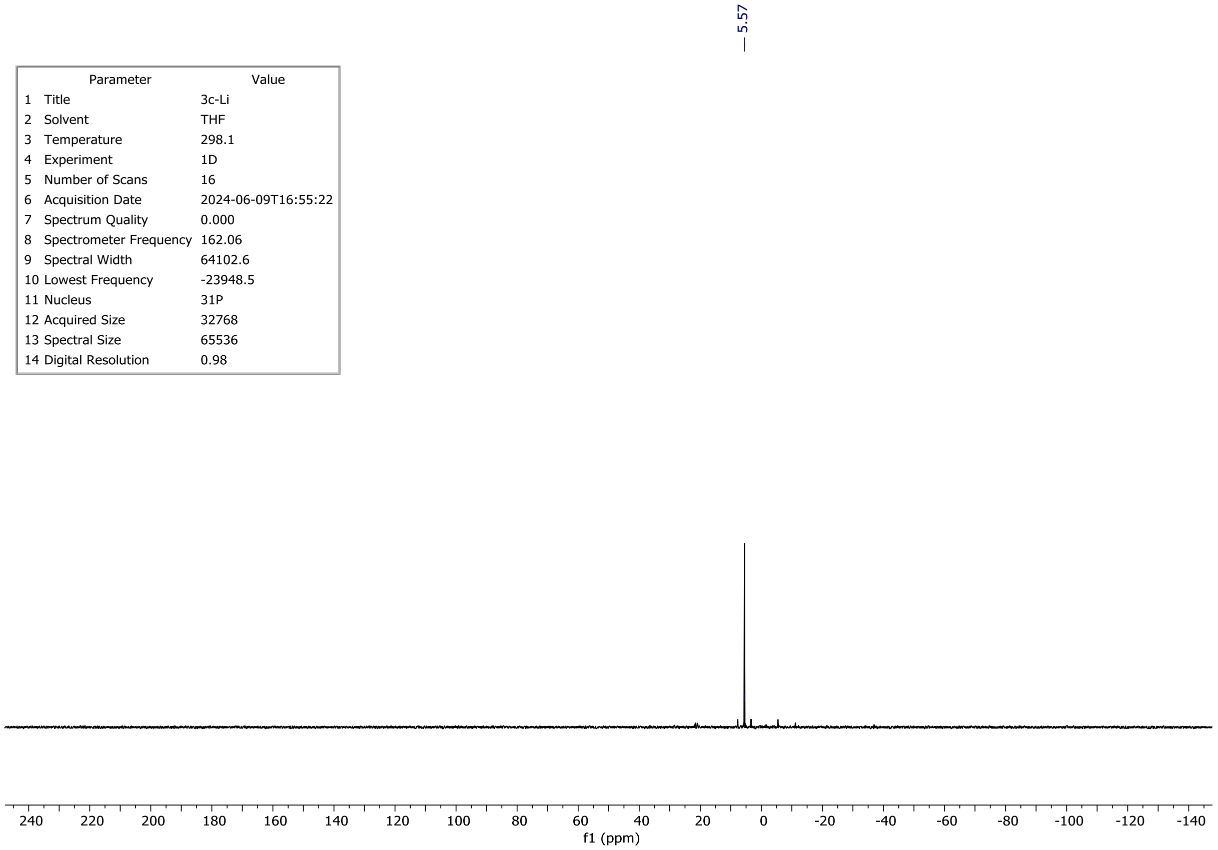

	Parameter	Value
1	Title	3c-Li
2	Solvent	THF
3	Temperature	298.1
4	Experiment	1D
5	Number of Scans	16
6	Acquisition Date	2024-06-09T16:55:22
7	Spectrum Quality	0.000
8	Spectrometer Frequency	162.06
9	Spectral Width	64102.6
10	Lowest Frequency	-23948.5
11	Nucleus	31P
12	Acquired Size	32768
13	Spectral Size	65536
14	Digital Resolution	0.98

Figure S27. $^{31}P\{^{1}H\}$ NMR spectrum of compound **3c-Li** in THF-d_8.

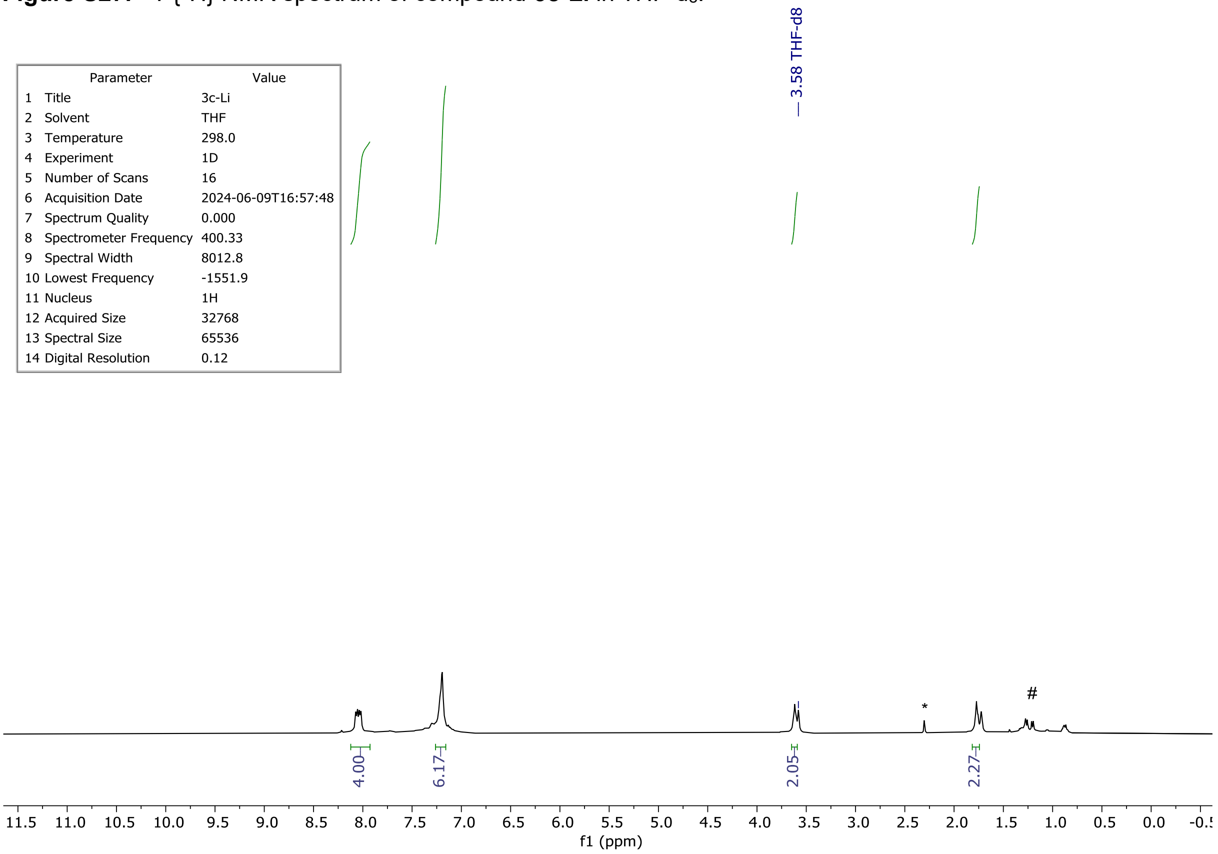

	Parameter	Value
1	Title	3c-Li
2	Solvent	THF
3	Temperature	298.0
4	Experiment	1D
5	Number of Scans	16
6	Acquisition Date	2024-06-09T16:57:48
7	Spectrum Quality	0.000
8	Spectrometer Frequency	400.33
9	Spectral Width	8012.8
10	Lowest Frequency	-1551.9
11	Nucleus	1H
12	Acquired Size	32768
13	Spectral Size	65536
14	Digital Resolution	0.12

Figure S28. ^{1}H NMR spectrum of compound **3c-Li** in THF-d_8. * corresponds to toluene and # corresponds to hexane.

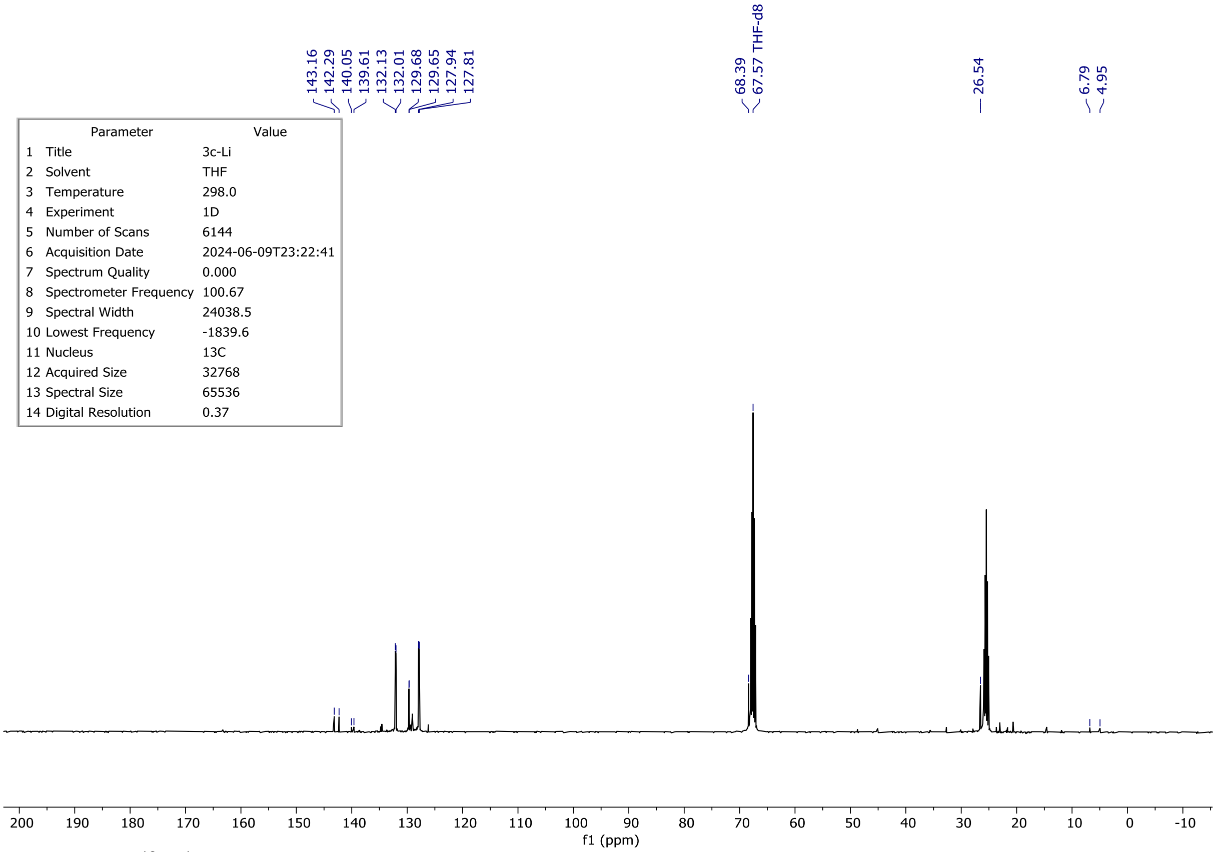

Figure S29. $^{13}C\{^{1}H\}$ NMR spectrum of compound **3c-Li** in THF-d_8.

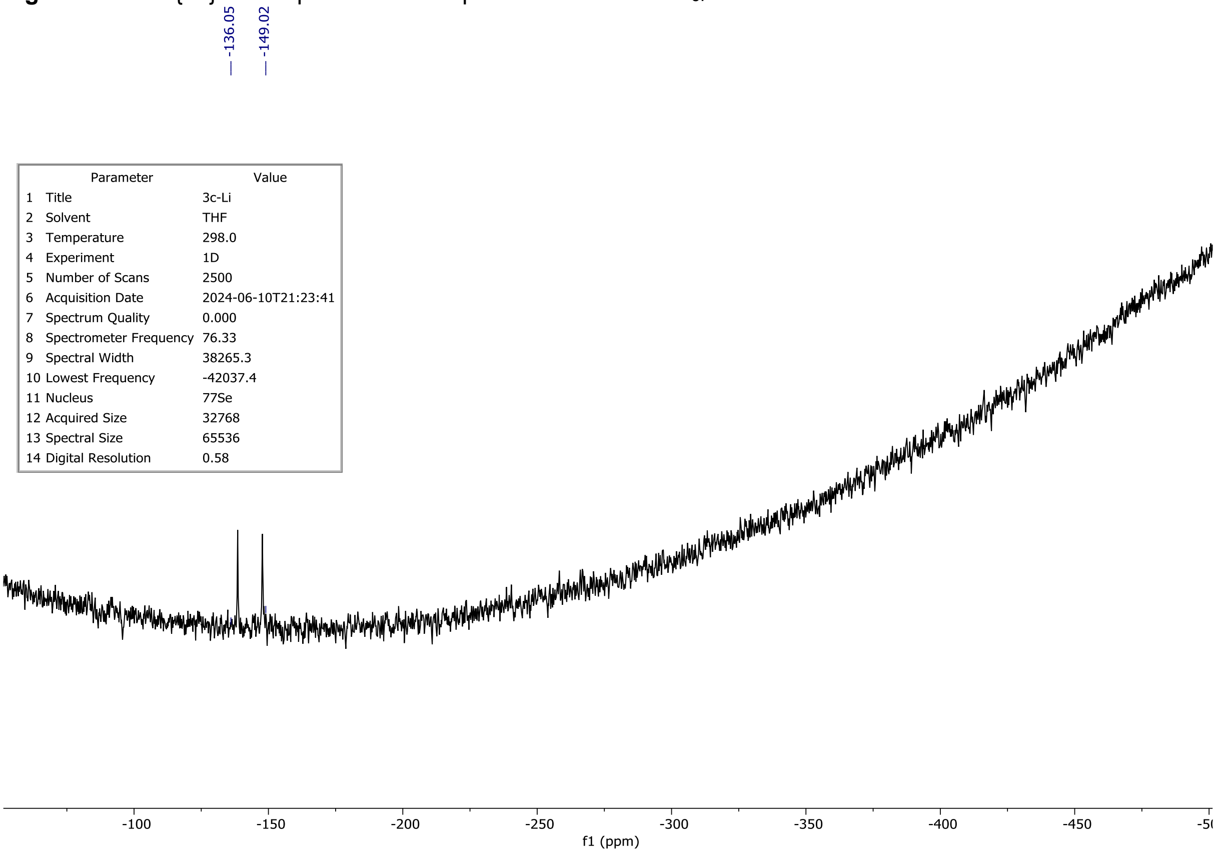

Figure S30. $^{77}Se\{^{1}H\}$ NMR spectrum of compound **3c-Li** in THF-d_8.

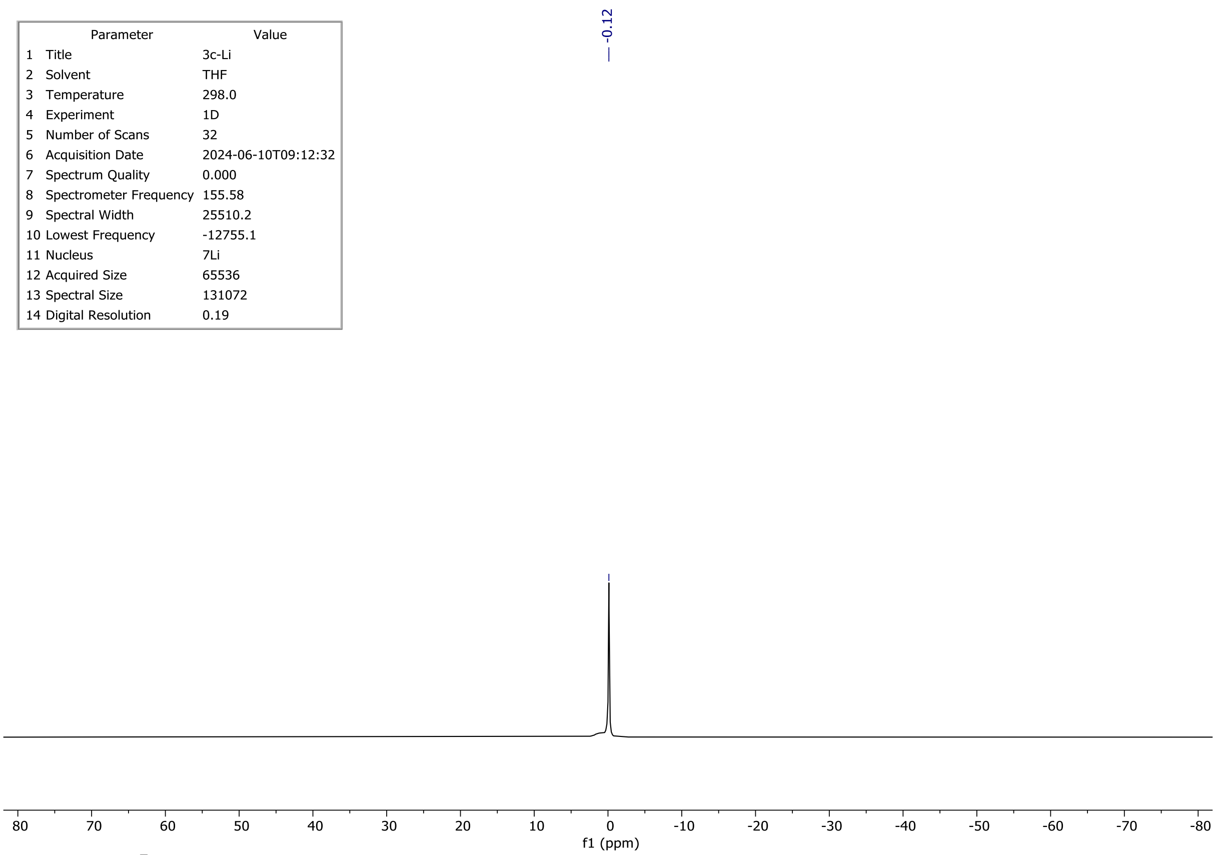

	Parameter	Value
1	Title	3c-Li
2	Solvent	THF
3	Temperature	298.0
4	Experiment	1D
5	Number of Scans	32
6	Acquisition Date	2024-06-10T09:12:32
7	Spectrum Quality	0.000
8	Spectrometer Frequency	155.58
9	Spectral Width	25510.2
10	Lowest Frequency	-12755.1
11	Nucleus	7Li
12	Acquired Size	65536
13	Spectral Size	131072
14	Digital Resolution	0.19

Figure S31. ^{7}Li NMR spectrum of compound **3c-Li** in THF-d_8.

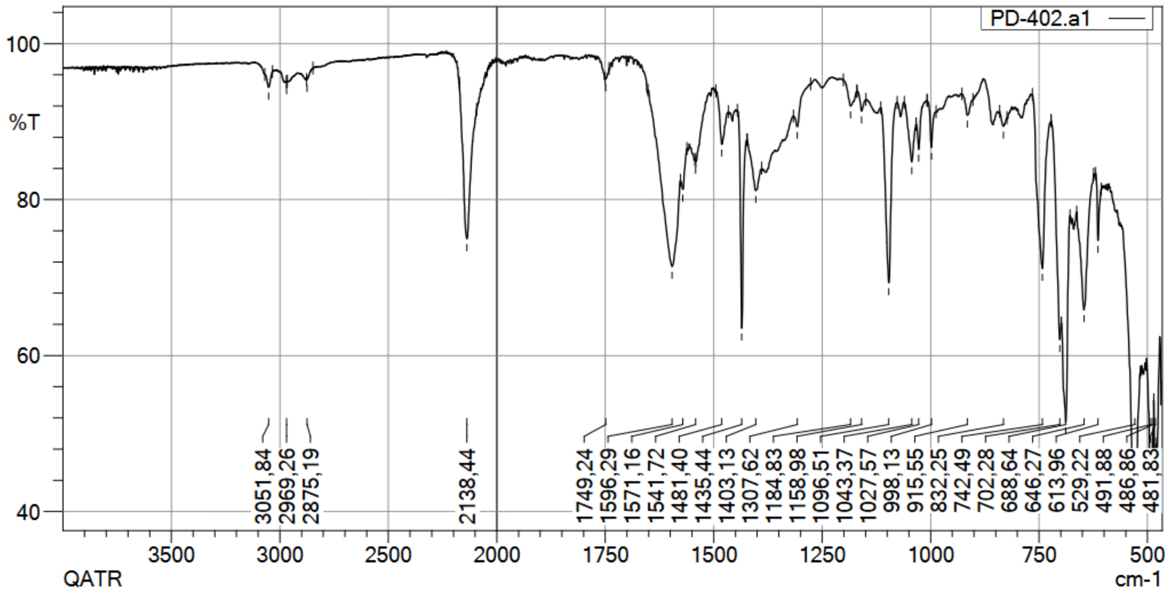

Figure S32. IR spectrum of compound **3c-Li** (solid state).

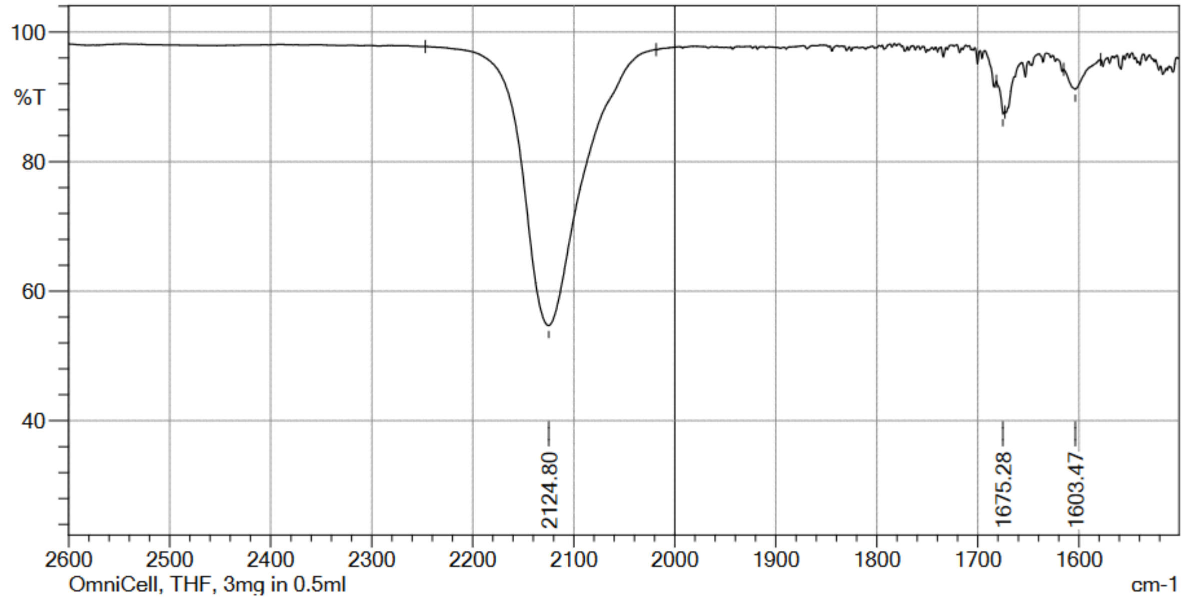

Figure S33. IR spectrum of compound **3c-Li** (THF solution).

	Parameter	Value
1	Title	3c-Li(PMDETA)
2	Solvent	THF
3	Temperature	295.2
4	Experiment	1D
5	Number of Scans	16
6	Acquisition Date	2024-02-03T08:54:09
7	Spectrum Quality	0.000
8	Spectrometer Frequency	162.06
9	Spectral Width	64102.6
10	Lowest Frequency	-23948.5
11	Nucleus	31P
12	Acquired Size	32768
13	Spectral Size	65536
14	Digital Resolution	0.98

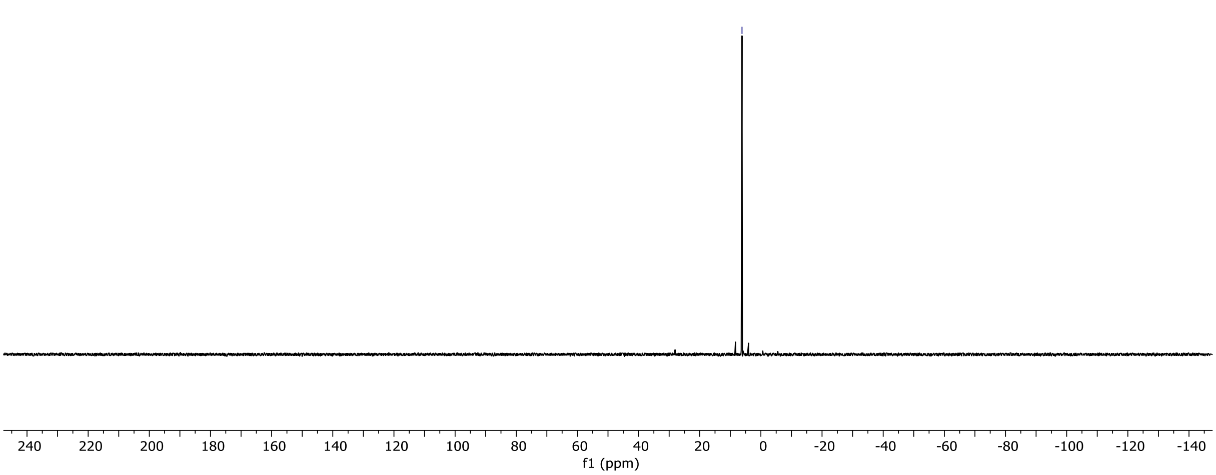

Figure S34. $^{31}P\{^{1}H\}$ NMR spectrum of compound **3c-Li**(PMDETA) in THF-d_8.

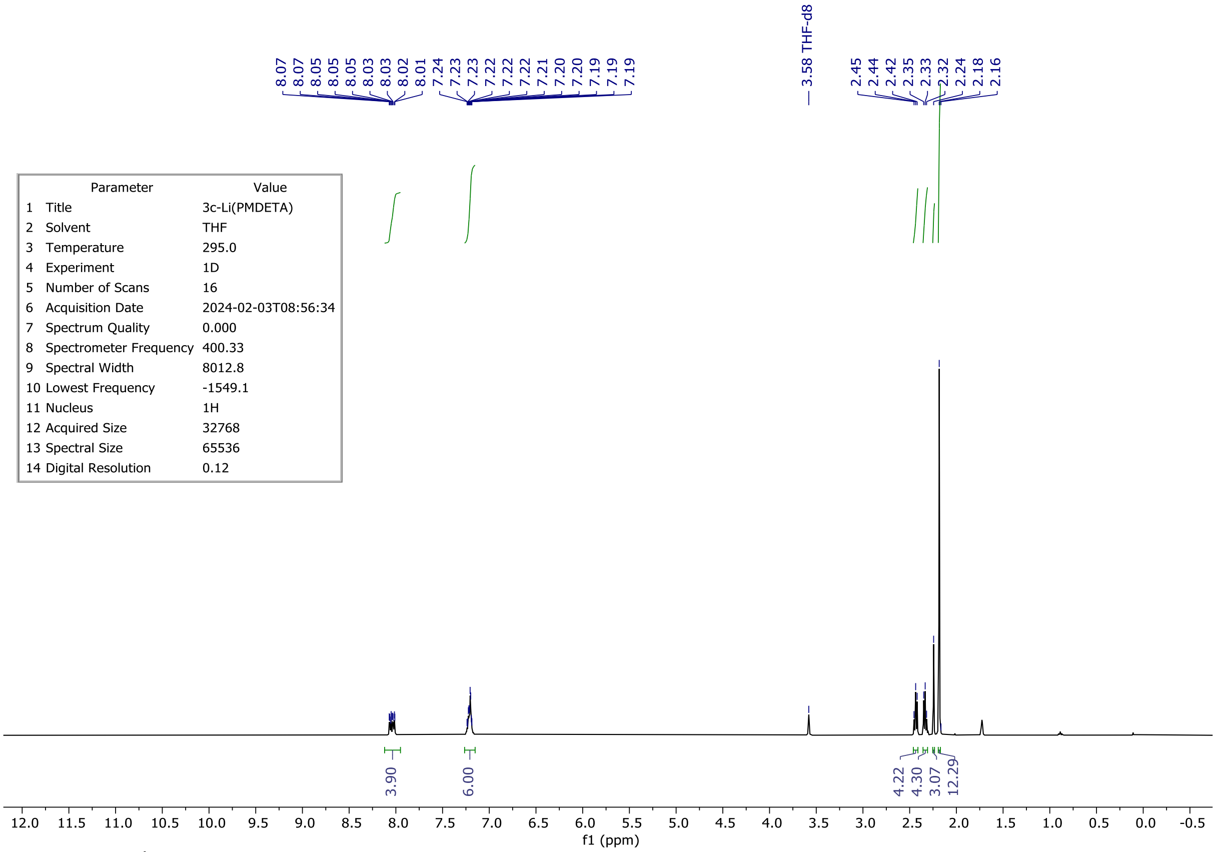

	Parameter	Value
1	Title	3c-Li(PMDETA)
2	Solvent	THF
3	Temperature	295.0
4	Experiment	1D
5	Number of Scans	16
6	Acquisition Date	2024-02-03T08:56:34
7	Spectrum Quality	0.000
8	Spectrometer Frequency	400.33
9	Spectral Width	8012.8
10	Lowest Frequency	-1549.1
11	Nucleus	1H
12	Acquired Size	32768
13	Spectral Size	65536
14	Digital Resolution	0.12

Figure S35. ^{1}H NMR spectrum of compound **3c-Li**(PMDETA) in THF-d_8.

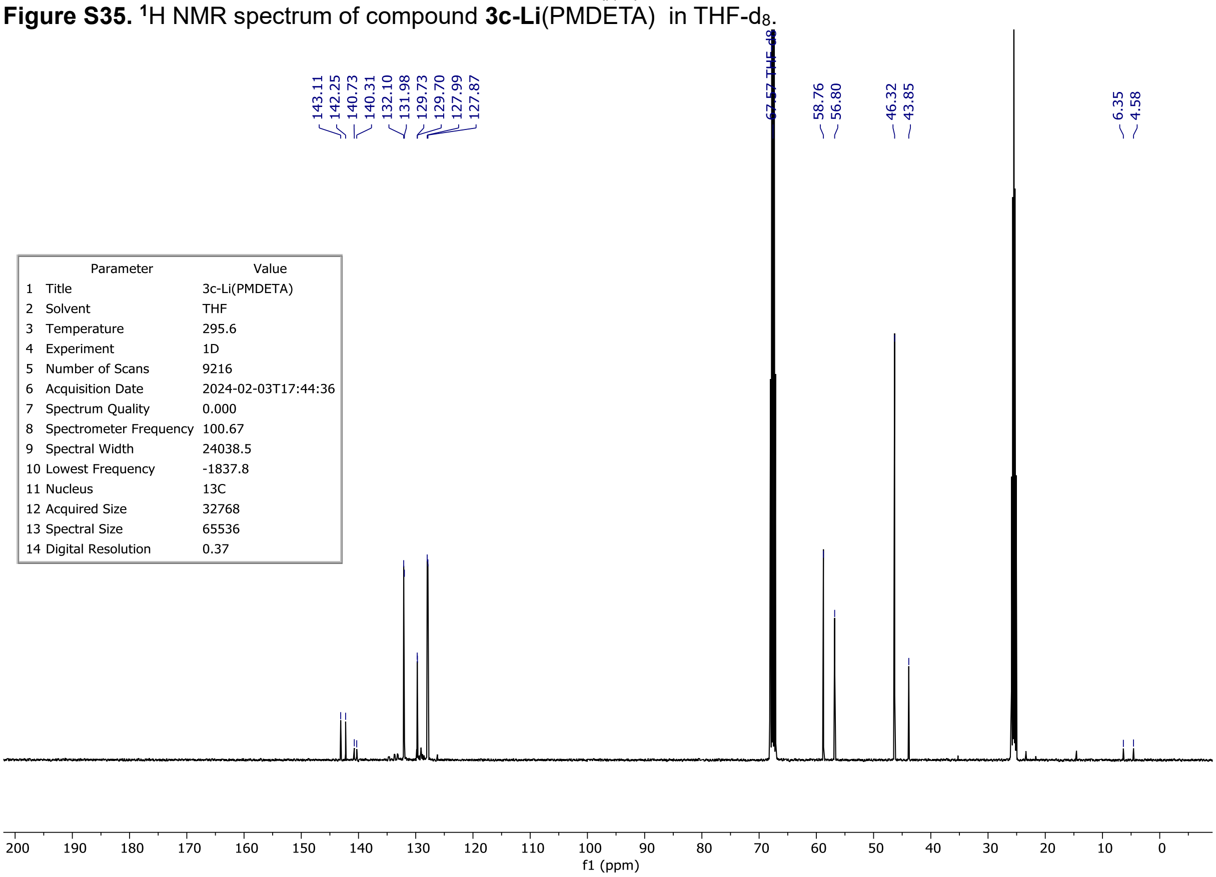

	Parameter	Value
1	Title	3c-Li(PMDETA)
2	Solvent	THF
3	Temperature	295.6
4	Experiment	1D
5	Number of Scans	9216
6	Acquisition Date	2024-02-03T17:44:36
7	Spectrum Quality	0.000
8	Spectrometer Frequency	100.67
9	Spectral Width	24038.5
10	Lowest Frequency	-1837.8
11	Nucleus	13C
12	Acquired Size	32768
13	Spectral Size	65536
14	Digital Resolution	0.37

Figure S36. $^{13}C\{^{1}H\}$ NMR spectrum of compound **3c-Li**(PMDETA) in THF-d_8.

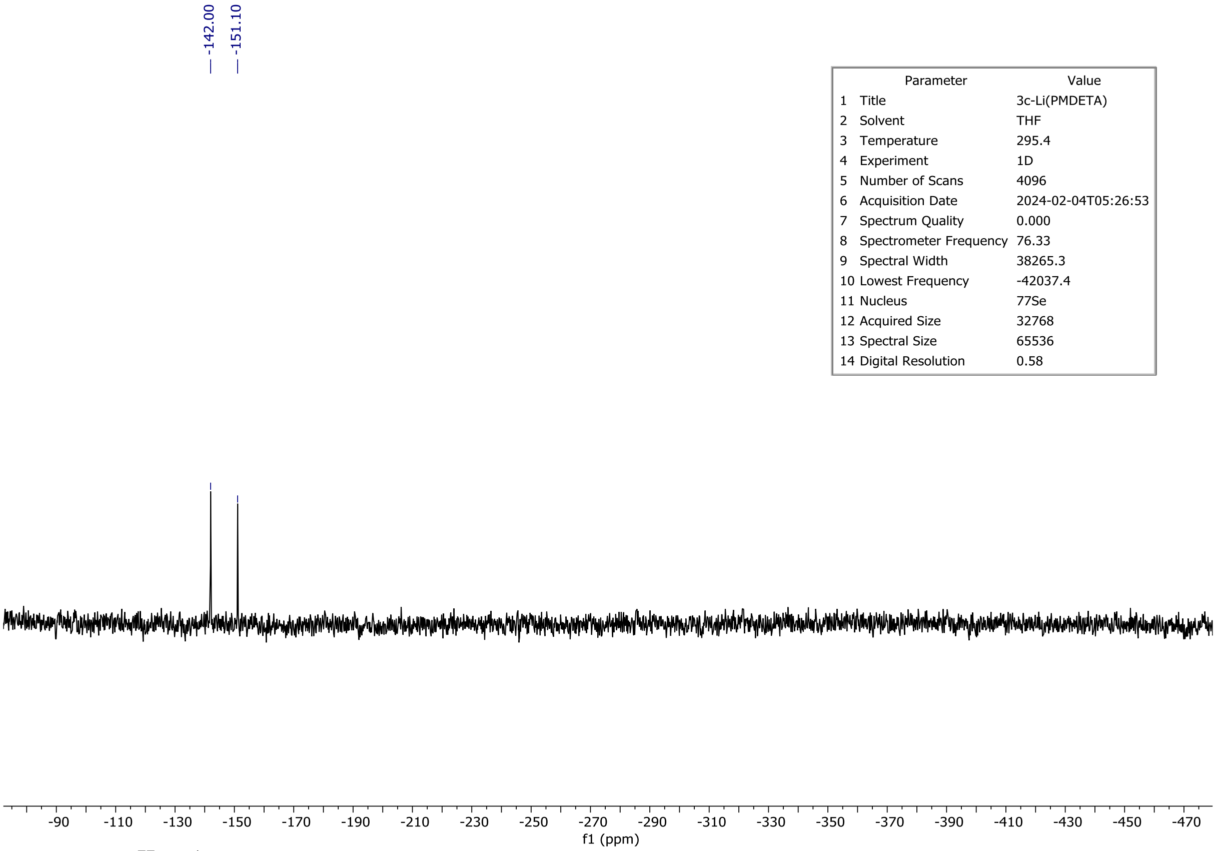

	Parameter	Value
1	Title	3c-Li(PMDETA)
2	Solvent	THF
3	Temperature	295.4
4	Experiment	1D
5	Number of Scans	4096
6	Acquisition Date	2024-02-04T05:26:53
7	Spectrum Quality	0.000
8	Spectrometer Frequency	76.33
9	Spectral Width	38265.3
10	Lowest Frequency	-42037.4
11	Nucleus	77Se
12	Acquired Size	32768
13	Spectral Size	65536
14	Digital Resolution	0.58

Figure S37. ^{77}Se{^{1}H} NMR spectrum of compound **3c-Li**(PMDETA) in THF-d_8.

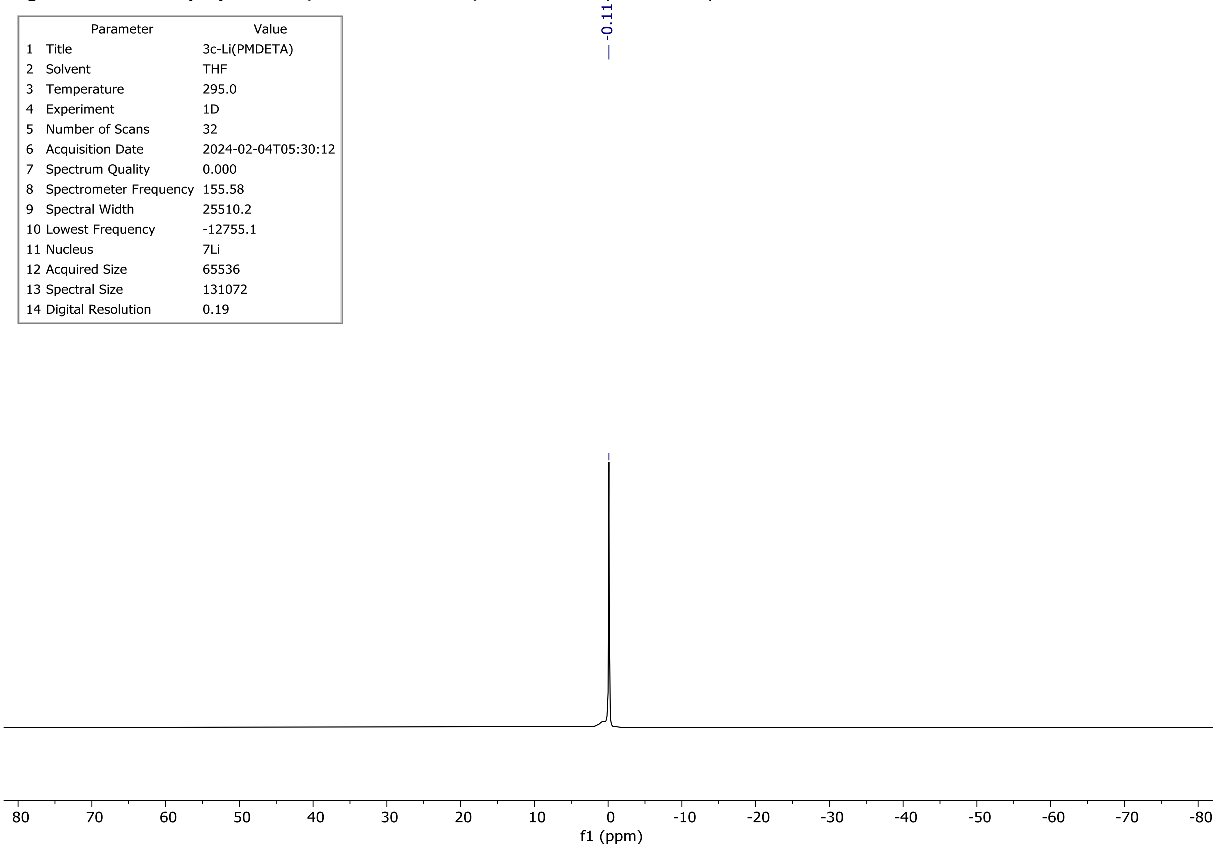

	Parameter	Value
1	Title	3c-Li(PMDETA)
2	Solvent	THF
3	Temperature	295.0
4	Experiment	1D
5	Number of Scans	32
6	Acquisition Date	2024-02-04T05:30:12
7	Spectrum Quality	0.000
8	Spectrometer Frequency	155.58
9	Spectral Width	25510.2
10	Lowest Frequency	-12755.1
11	Nucleus	7Li
12	Acquired Size	65536
13	Spectral Size	131072
14	Digital Resolution	0.19

Figure S38. ^{7}Li NMR spectrum of compound **3c-Li**(PMDETA) in THF-d_8.

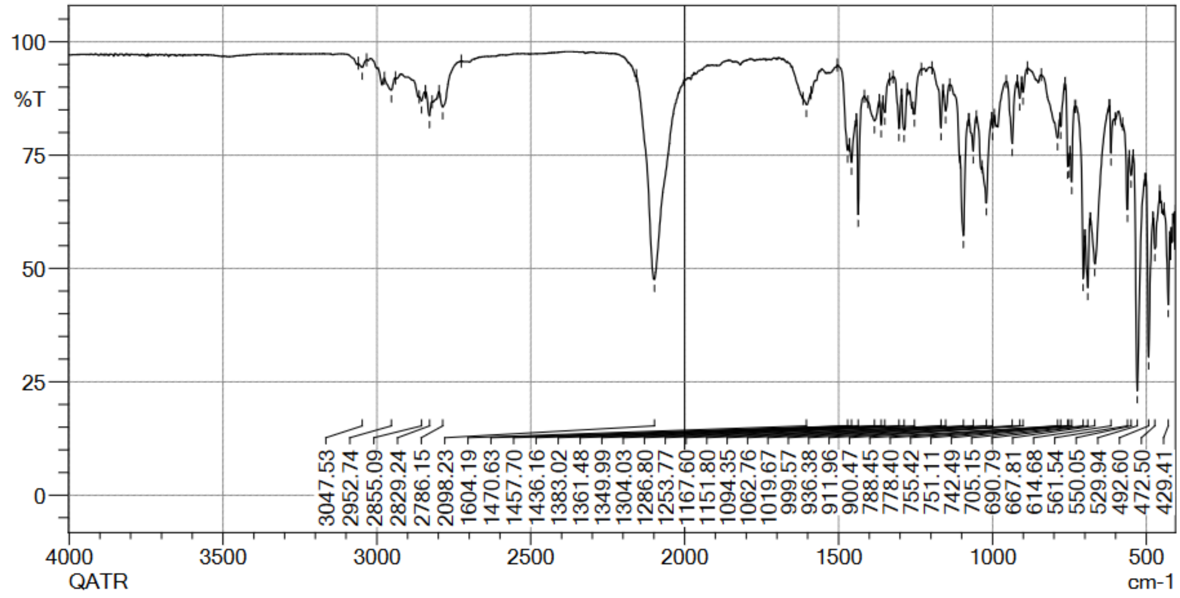

Figure S39. IR spectrum of compound **3c-Li** (PMDETA) (solid state).

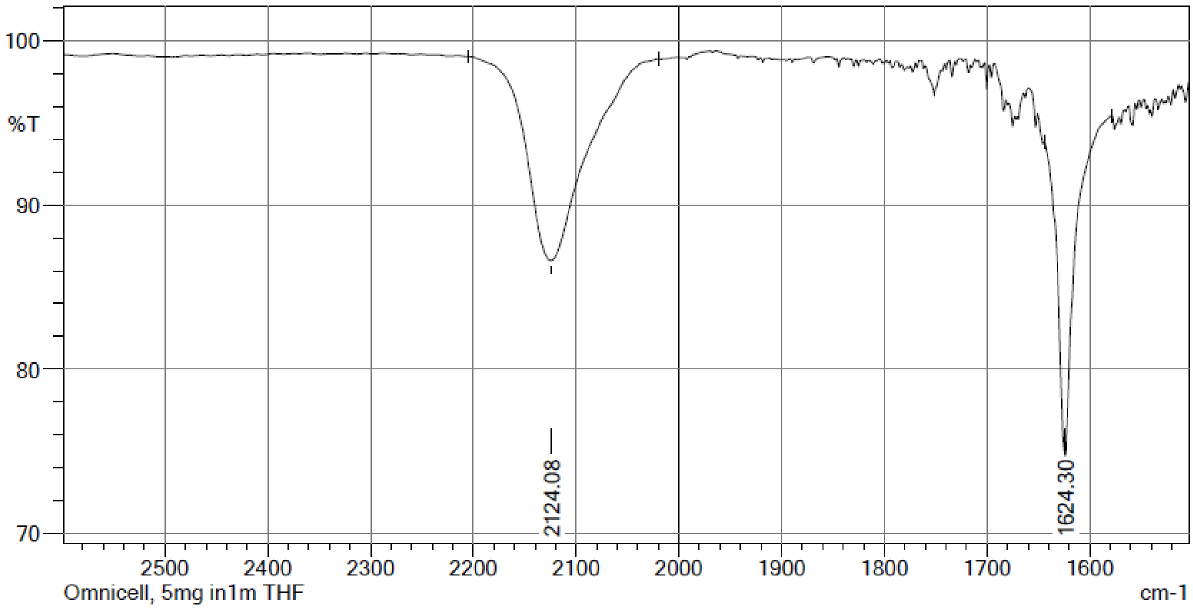

Figure S40. IR spectrum of compound **3c-Li** (PMDETA) (solid state).

3. Crystal structure determination

3.1. General information

Good quality single crystals were hand-picked under polarized optical microscopy and then mounted on the diffractometer. The data collection was done at 100 K. X-ray intensity data measurements of **all compounds** were carried out on an Oxford SuperNova diffractometer with graphite-monochromatized (CuK_{α}= 1.54184 Å) radiation. The X-ray generator was operated at 50 kV and 30 mA. All the structures were solved using direct methods, refined with the Shelx software package[3-6] and expanded using Fourier techniques. The crystals of all compounds were mounted in an inert oil such as perfluoropolyalkylether. Crystallographic data including structure factors have been deposited with the Cambridge Crystallographic Data Centre as supplementary publication no. CCDC- 236206 to CCDC-236209 Copies of the data can be gained free of charge on application to Cambridge Crystallographic Data Centre, 12 Union Road, Cambridge CB2 1EZ, UK; [fax: (+44) 1223-336-033; email: deposit@ccdc.cam.ac.uk].

Table S1. Data collection and structure refinement details for compounds **2b** and **2c.**

**Compound**	**2b**	**2c**
Formula	C70 H66 Li2 O2 P4 S2	C70 H66 Li2 O2 P4 Se2
CCDC	2362607	2362606
Formula weight	1141.10	1234.90
Temperature [K]	100(2)	100(2)
Wave lenght [Å]	1.54184	1.54184
Crystal system	Monoclinic	Monoclinic
Space group	$P2_1/c$	$P2_1/c$
a [Å]	14.7042(2)	15.72650(10)
b [Å]	11.10570	9.50190(10)
c [Å]	18.8325(2)	20.4394(2)
α[°]	90	90
β [°]	106.0010(10)	97.1570(10)
γ [°]	90	90
Volumen [$Å^3$]	2956.21(6)	3030.50(5)
Z	2	2
Calc. density [$Mg{\cdot}m^{-3}$]	1.282	1.353
μ ($Mo_{K\alpha}$) [mm^{-1}]	2.193	2.869
F(000)	1200	1272
Crystal dimensions [mm]	0.360 x 0.310 x 0.080	0.280 x 0.200 x 0.140
Theta range θ [°]	3.127 to 67.997	2.832 to 76.746

Index ranges	-17<=h<=16<br>-13<=k<=13<br>-22<=l<=22	-19<=h<=19<br>-10<=k<=11<br>-25<=l<=25
Reflections collected	35590	38788
Independent reflections	5382 [R(int) = 0.0413]	6214 [R(int) = 0.0445]
Data/Restraints/Parameter	5382 / 0 / 361	6214 / 0 / 361
Goodness-of-fit on F^2	1.016	1.045
Final R indices [I>2sigma(I)]	R1 = 0.0343, wR2 = 0.0900	R1 = 0.0343, wR2 = 0.0879
Largest diff. peak and hole [e·Å-3]	0.486 and -0.407	1.184 and -0.595

Table S1. Data collection and structure refinement details for compounds **3a-Li** and **3c-Li**(PMDETA).

**Compound**	**3a-Li**	**3c-Li**(PMDETA)
Formula	C36 H36 Li2 O6 P2	C46 H66 Li2 N6 O2 P2 Se2
CCDC	2362609	2362608
Formula weight	640.47	968.78
Temperature [K]	293(2)	100(2)
Wave lenght [Å]	1.54184	1.54184
Crystal system	Triclinic	Monoclinic
Space group	P-1	C2
a [Å]	14.2502(3)	23.8309(5)
b [Å]	15.4854(3)	9.4168(2)
c [Å]	15.5126(3)	11.2449(2)
α[°]	90.527(2)	90
β [°]	91.990(2)	103.333(2)
γ [°]	100.300(2)	90
Volume [$Å^3$]	3365.56(12)	2455.46(9)
Z	4	2
Calc. density [$Mg{\cdot}m^{-3}$]	1.264	1.310
μ ($Mo_{K\alpha}$) [mm^{-1}]	1.528	2.818
F(000)	1344	1008
Crystal dimensions [mm]	0.313 x 0.144 x 0.136	0.250 x 0.190 x 0.090
Theta range θ [°]	2.851 to 76.831	3.812 to 76.642
Index ranges	-17<=h<=17<br>-19<=k<=19<br>-17<=l<=19	-26<=h<=30<br>-11<=k<=11<br>-14<=l<=13

Reflections collected	22471	12718
Independent reflections	22471 [R(int) = 0.0564]	4616 [R(int) = 0.0454]
Data/Restraints/Parameter	22471 / 0 / 830	4616 / 1 / 276
Goodness-of-fit on F^2	1.157	1.049
Final R indices [I>2sigma(I)]	R1 = 0.0660, wR2 = 0.1833	R1 = 0.0552, wR2 = 0.1450
Largest diff. peak and hole [e·Å-3]	0.672 and -0.537	2.188 and -0.715

3.2. Molecular structure of 2b

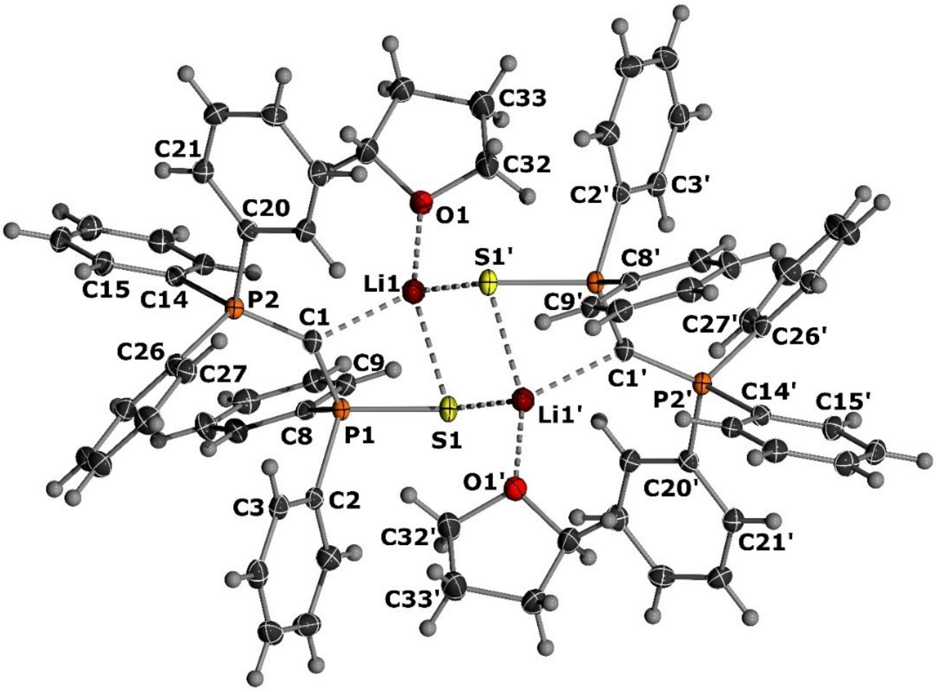

Figure S41. Molecular structure of compound **2b**. Thermal ellipsoids at 50% probability level. Selected bond lengths [Å] and angles [°]: P1-C1 1.671(2), P2-C1 1.651(2), P1-S1 2.0296(5), C1-Li1 2.128(3), S1-Li1 2.558(3), P1-C1-P2 135.86(10).

3.3. Molecular structure of 2c

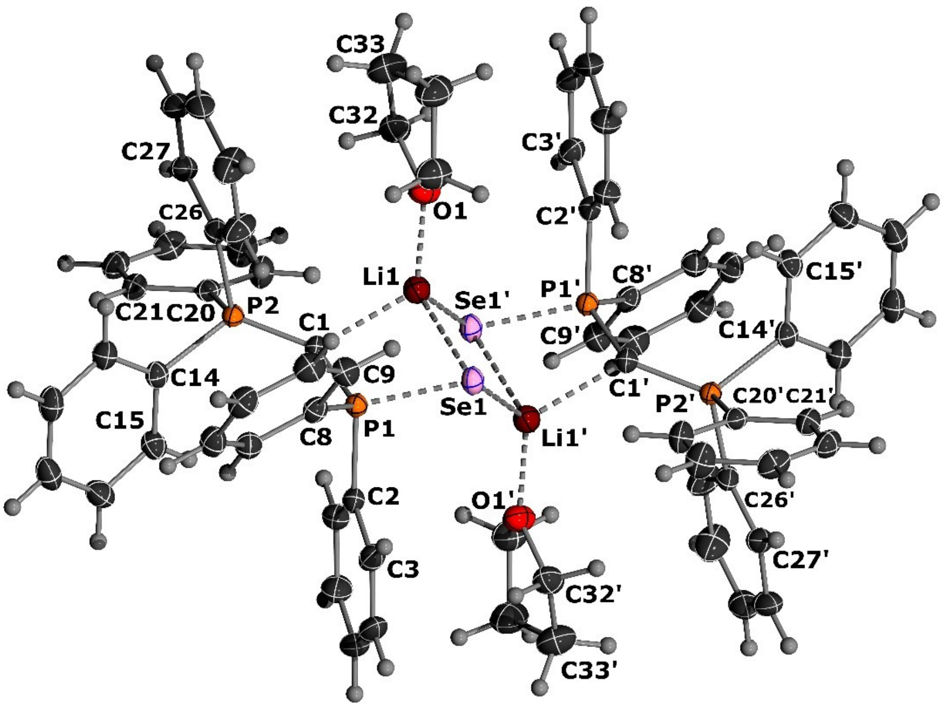

Figure S42. Molecular structure of compound **2b**. Thermal ellipsoids at 50% probability level. Selected bond lengths [Å] and angles [°]: P1-C1 1.674(2), P2-C1 1.646(2), P1-Se1 2.193(5), C1-Li1 2.159(4), Se1-Li1 2.622(4), P1-C1-P2 132.04(12).

3.4. Molecular structure of 3a-Li

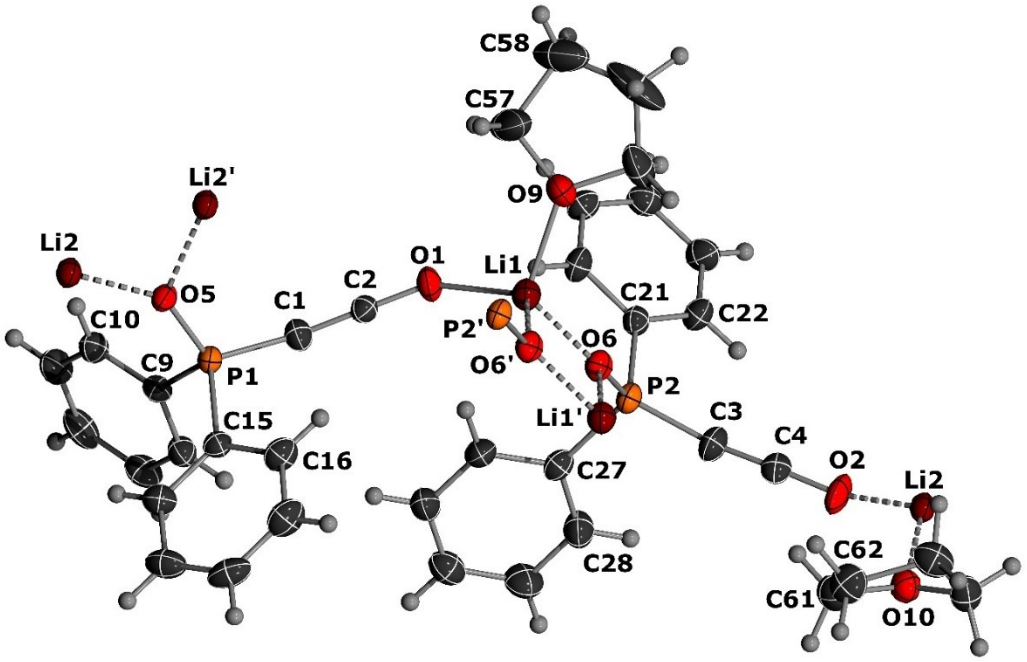

Figure S43. Molecular structure of compound **3a-Li**. Thermal ellipsoids at 50% probability level. Selected bond lengths [Å] and angles [°]: P1-C1 1.677(4), C1-C2 1.230(6), C2-O1 1.216(5), P1-O1 1.510(3), P1-C1-C2 161.9(4), C1-C2-O1 176.7(5), P2-C3 1.673(4), C3-C4 1.231(6), C4-O2 1.206(5), P2-O6 1.509(3), P2-C3-C4 162.4(4), C1-C2-O1 176.7(5).

3.5. Molecular structure of 3c-Li(PMDETA)

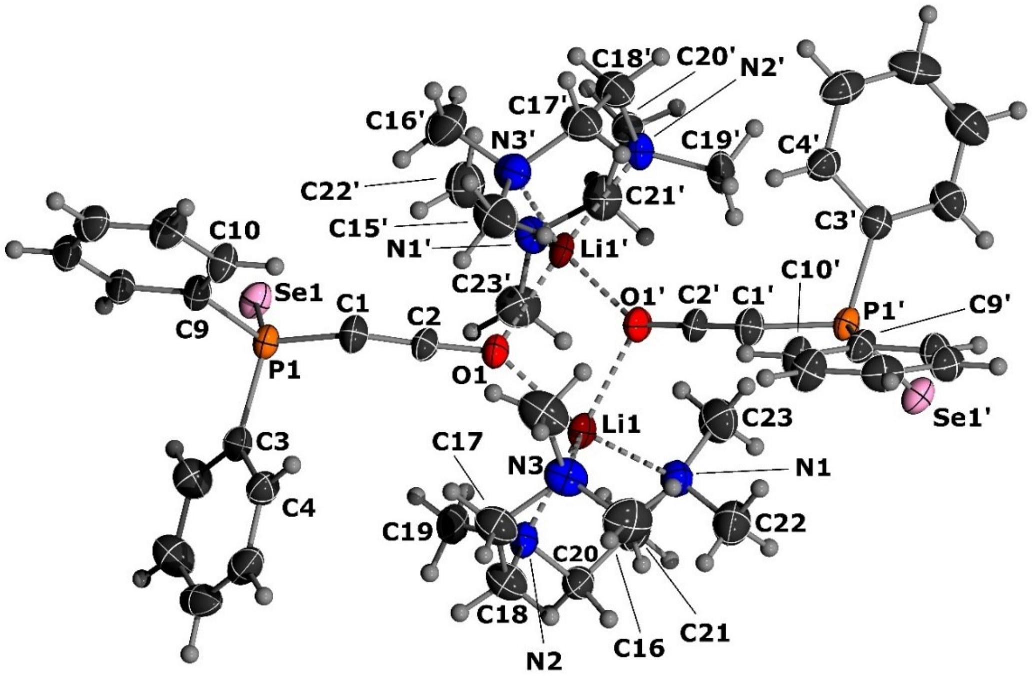

Figure S44. Molecular structure of compound **3c-Li**(PMDETA). Thermal ellipsoids at 50% probability level. Selected bond lengths [Å] and angles [°]: P1-Se1 2.132(1), P1-C1 1.698(6), C1-C2 1.243(8), C2-O1 1.222(7), O1-Li1 2.069(10), P1-C1-C2 147.8(5), C1-C2-O1 173.9(6).

3.6. Molecular structure of 4c

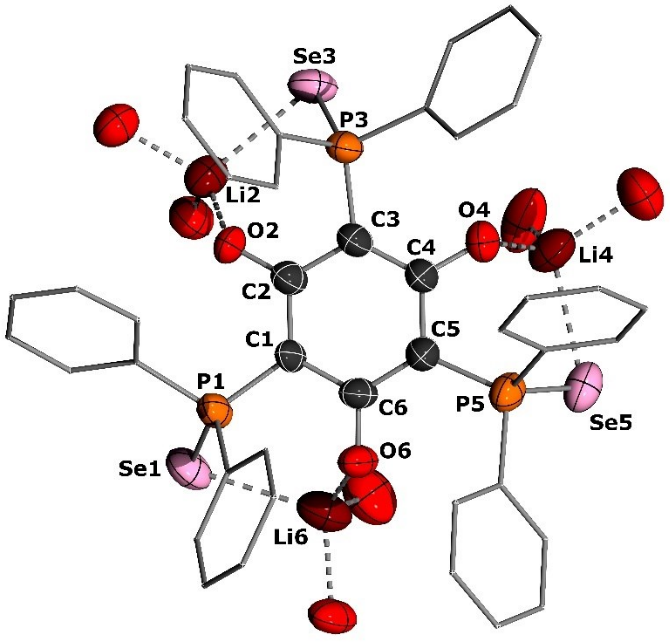

Figure S45. Molecular structure of compound **4c**. Thermal ellipsoids at 50% probability level. H atoms and THF molecules are omitted for clarity. The bond lengths and bond angles are not discussed due to the low quality of the crystal.

3.7. Molecular structure of potassium ketenyl anions

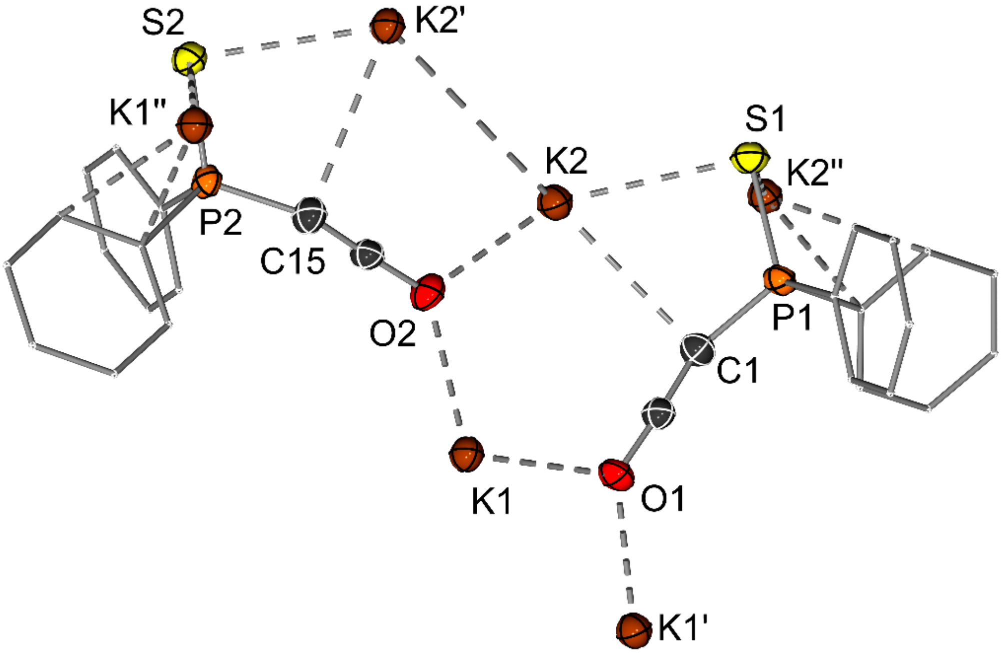

Figure S46. Molecular structure of **3b-K**. (CCDC-2201263) Thermal ellipsoids at 50% probability level. Selected bond lengths [Å] and angles [°]: P1-C1 1.695(6), C1-C2 1.240(8), C2-O1 1.216(7), P1-C1-C2 145.6(6), C1-C2-O1 175.0(7). This structure has been reported in reference 1.

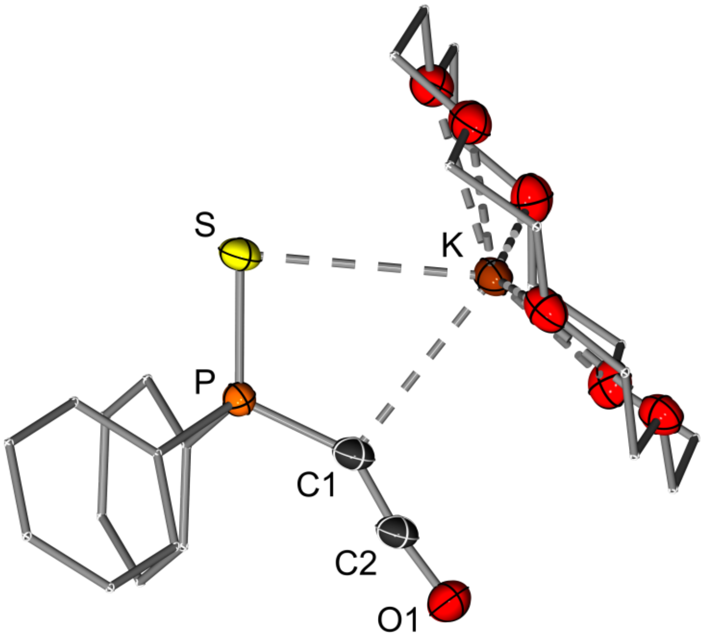

Figure S47. Molecular structure of **3b-K(18-C-5)**. (CCDC-2201261) Thermal ellipsoids at 50% probability level. Selected bond lengths [Å] and angles [°]: P1-C1 1.698(3), C1-C2 1.178(8), C2-O1 1.248(8), C1-K1 3.056(3), P1-C1-C2 146.2(5), C1-C2-O1 174.5(11). This structure has been reported in reference 1.

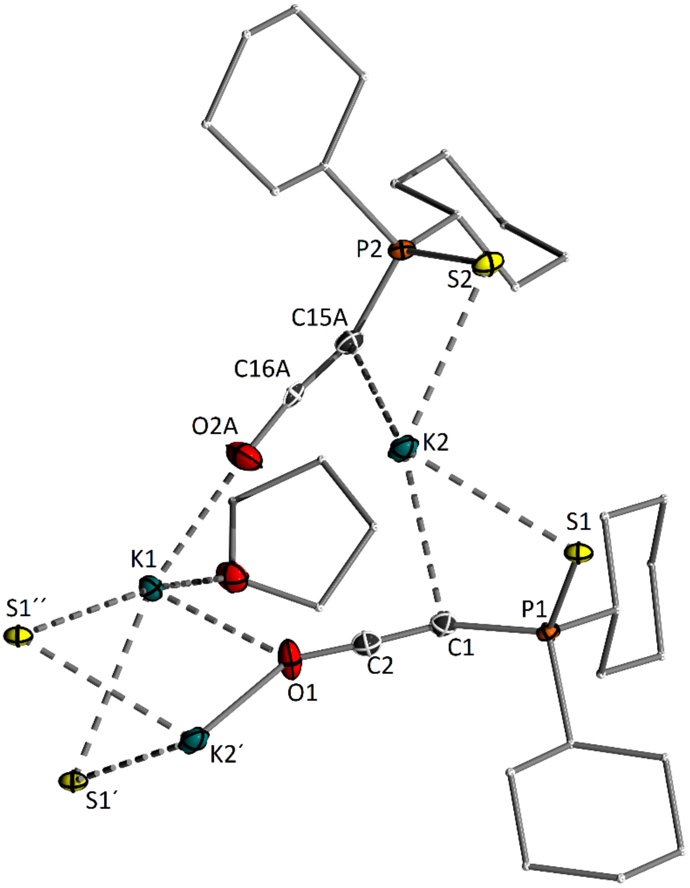

Figure S48. Molecular structure of **3b^{Cy}-K.** (CCDC-2320773) Thermal ellipsoids at 50% probability level. Selected bond lengths [Å] and angles [°]: C1–C2 1.235(4), C2–O1 1.217(3), P–C1 1.704(3), P–S 2.007(1), P–C1–C2 150.9(2), C1–C2–O1 175.5(3). This structure has been reported in reference 2.

4. Author contributions

P.D. and S.M. carried out the synthetic work and standard analytical characterization. P.D. conducted the XRD analyses. M.J. prepared one starting material. V.H.G. has designed the study, supervised the research activities, and prepared the manuscript together with P.D. and the help of S.M.

5. References

1. M. Jorges, F. Krischer and V. H. Gessner, *Science* 2022, **378**, ,1331−1336.
2. M. Jörges, S. Mondal, M. Kumar, P. Duari, F. Krischer, J. Löffler and V. H. Gessner, Organometallics 2024, **43**, 585−593
3. G. M. Sheldrick, A short history of SHELX. Acta Cryst A 2008, **64 (Pt 1)**, 112–122. DOI: 10.1107/S0108767307043930. Published Online: Dec. 21, 2007.
4. G. M. Sheldrick, Crystal structure refinement with SHELXL. Acta Cryst C 2015, **71 (Pt 1)**, 3–8. DOI: 10.1107/S2053229614024218. Published Online: Jan. 1, 2015.
5. G. M. Sheldrick, SHELXT - integrated space-group and crystal-structure determination. Acta Cryst A 2015, **71 (Pt 1)**, 3–8. DOI: 10.1107/S2053229614024218. Published Online: Jan. 1, 2015.
6. A. Thorn, B. Dittrich, G. M. Sheldrick, Enhanced rigid-bond restraints. Acta Cryst A 2012, **68 (4)**, 448–451. DOI: 10.1107/S0108767312014535.
